# Abnormal patterns of sleep and waking behaviors are accompanied by neocortical oscillation disturbances in an *Ank3* mouse model of epilepsy-bipolar disorder comorbidity

**DOI:** 10.1038/s41398-023-02700-2

**Published:** 2023-12-20

**Authors:** Juan E. Villacres, Nicholas Riveira, Sohmee Kim, Laura L. Colgin, Jeffrey L. Noebels, Angel Y. Lopez

**Affiliations:** 1 Center for Learning and Memory, The University of Texas at Austin, Austin, TX 78712-0805 USA; 2 Department of Neuroscience, The University of Texas at Austin, Austin, TX 78712-0805 USA; 3 Department of Biomedical Engineering, The University of Texas at Austin, Austin, TX 78712-0805 USA; 4 Department of Electrical and Computer Engineering, The University of Texas at Austin, Austin, TX 78712-0805 USA; 5 Institute for Neuroscience, The University of Texas at Austin, Austin, TX 78712-0805 USA; 6 Department of Neurology, Baylor College of Medicine, Houston, TX 77030 USA; 7 Department of Neuroscience, Baylor College of Medicine, Houston, TX 77030 USA; 8 Department of Molecular and Human Genetics, Baylor College of Medicine, Houston, TX 77030 USA

**Keywords:** Physiology, Neuroscience, Bipolar disorder

## Abstract

*ANK3* is a leading bipolar disorder (BD) candidate gene in humans and provides a unique opportunity for studying epilepsy-BD comorbidity. Previous studies showed that deletion of *Ank3-1b*, a BD-associated variant of *Ank3* in mice leads to increased firing threshold and diminished action potential dynamic range of parvalbumin (PV) interneurons and absence epilepsy, thus providing a biological mechanism linking epilepsy and BD. To explore the behavioral overlap of these disorders, we characterized behavioral patterns of *Ank3-1b* KO mice during overnight home-cage activity and examined network activity during these behaviors using paired video and EEG recordings. Since PV interneurons contribute to the generation of high-frequency gamma oscillations, we anticipated changes in the power of neocortical EEG signals in the gamma frequency range (> 25 Hz) during behavioral states related to human BD symptoms, including abnormal sleep, hyperactivity, and repetitive behaviors. *Ank3-1b* KO mice exhibited an overall increase in slow gamma (~25-45 Hz) power compared to controls, and slow gamma power correlated with seizure phenotype severity across behaviors. During sleep, increased slow gamma power correlated with decreased time spent in the rapid eye movement (REM) stage of sleep. Seizures were more common during REM sleep compared to non-REM (NREM) sleep. We also found that *Ank3-1b* KO mice were hyperactive and exhibited a repetitive behavior phenotype that co-occurred with increased slow gamma power. Our results identify a novel EEG biomarker associating *Ank3* genetic variation with BD and epilepsy and suggest modulation of gamma oscillations as a potential therapeutic target.

## Introduction

Epilepsy and bipolar disorder (BD) are chronic, episodic, and debilitating disorders that often lead to suicide. Epidemiological studies suggest that approximately 10% of people with epilepsy experience symptoms of BD, including mania, depression, and disrupted patterns of sleep [1]. Reciprocally, people with BD are at a four-fold increased risk of developing epilepsy [2–4]. In addition to these shared features and bidirectional comorbidity, anticonvulsant medications are often used to effectively treat BD, and kindling effects seen in BD are hypothesized to be related to kindling in epilepsy [5]. These data suggest that these two disorders share mechanistic links. However, identifying such mechanisms has remained a challenge [6].

The *ANK3* gene provides a singular opportunity to explore the nexus of these disorders. *ANK3* is a leading BD candidate gene with links to epilepsy [7]. Rare variants of significant effect in coding regions of *ANK3* have been identified in families with BD and mood-related psychiatric symptoms [8–10], and common variants in noncoding regions of *ANK3* are more widely associated with BD through multiple, independent genome-wide association studies (GWAS) [11–16]. These GWAS show a hotspot for single nucleotide polymorphisms (SNPs) in an intronic, candidate cis-regulatory region upstream of *ANK3*’s third alternative-first-exon (exon-1b). Exon-1b and its upstream cis-regulatory sequence is highly conserved among vertebrates, and ChIP-seq data shows similar transcription factor binding affinities for these regions between rodents and humans (https://genome.ucsc.edu/index.html) [17, 18]. Furthermore, human studies suggest that SNPs in this region are associated with reduced expression of *ANK3* transcripts in patients with BD and accompanied by altered neuropathology [19–21]. A recent study identified a female patient with a novel homozygous missense variant (c.178 T > C; p.Tyr60His) in exon-1b of the *ANK3* gene who exhibited seizures and mood disturbance [22].

*ANK3* is a very large gene that undergoes significant alternative splicing to produce various isoforms of the scaffolding protein, ankyrin-G (ankG). *ANK3* has three alternative-first-exons, two of which are utilized by neurons and glia in the brain (exon-1e and exon-1b) to transcribe three main groups of ankG isoforms with different functions. Members of the 480 kDa group of isoforms are found at GABAergic synapses and the axon initial segment (AIS) of neurons [23]. At the AIS, exon-1e and exon-1b code for alternative N-termini (NT2 and NT3, respectively) of AnkG, which serve to regulate the binding of sodium and potassium channels to the adjacent membrane binding domain [24].

Our previous study discovered that GABAergic parvalbumin (PV) interneurons exclusively express NT3-AnkG isoforms at the AIS, while excitatory pyramidal cells in many brain regions express NT2-ankG. This work showed that PV interneurons in *Ank3-exon1b* (*Ank3-1b*) knockout (KO) mice have a reduced number of voltage-gated sodium channels at the AIS, increased firing threshold, and diminished action potential dynamic firing range at frequencies above 100 Hz [7]. Additionally, this work showed that *Ank3-1b* deletion results in reduced cortical network inhibition and an absence epilepsy phenotype that was recapitulated by knocking out all ankG isoforms selectively in PV interneurons. This makes *Ank3-1b* mice a valuable and so far unique model for studying monogenic epilepsy-BD comorbidity. These findings led to the hypothesis that imbalanced excitation and inhibition due to PV interneuron dysfunction may underlie the genetic association between *ANK3* and BD and serve as a shared mechanistic link connecting thalamocortical epilepsy and mood disorder.

PV interneuron deficiencies and aberrant gamma rhythms have been implicated in BD through human studies [25–29], and a familial mutation of *ANK3* (W1989R) caused disrupted cortical and hippocampal gamma oscillations in a knock-in mouse model [9]. The fast-spiking properties of PV interneurons are important for generating and modulating high-frequency gamma oscillations [30]. Thus, we hypothesized changes in the power of gamma oscillations in *Ank3-1b* KO mice. Additionally, we hypothesized that changes in gamma power in this model would be accompanied by changes in behaviors relevant to epilepsy-BD comorbidity, such as sleep disturbance and altered activity levels.

Previous behavioral studies of *Ank3-1b* heterozygous (*Ank3-1b*^*+/KO*^) KO mice have been used to model BD. In one study, a battery of behavioral assays was used to phenotype *Ank3-1b*^*+/KO*^ mice and found decreased anxiety, increased motivation for reward at baseline, and a transition to depression-related features after chronic stress via isolation [31]. However, the effect sizes were relatively small, making reproducibility a challenge for in vivo electrophysiological studies that are often limited in cohort size due to technical and time constraints. Also, brief behavioral assays may not capture mood oscillations congruent with those of BD patients, which require study over longer periods of time.

One way to address these issues is to utilize recent high-throughput methods of monitoring rodent behavior over prolonged uninterrupted periods of home cage activity, allowing the study of some common endophenotypes of BD that have not yet been explored in this model, such as sleep disturbances and repetitive behaviors. In the present study, we adopted an automated approach to behaviorally phenotype *Ank3-1b* KO mice using the open-source learning algorithm, DeepLabCut [7], while simultaneously examining gamma oscillations using paired video-EEG recordings taken from the *Ank3-1b* KO mice and their littermate controls (Supplementary Fig. 1).

## Materials and Methods

All experimental procedures and data, including Supplementary Figs. 1–14 are included in the article and in Supplementary information. Materials and protocols are available upon request from the corresponding author (A.Y.L.).

## Results

### Increased slow gamma power in *Ank3-1b* KO mice correlated with epilepsy phenotype severity

**Fig. 1:**
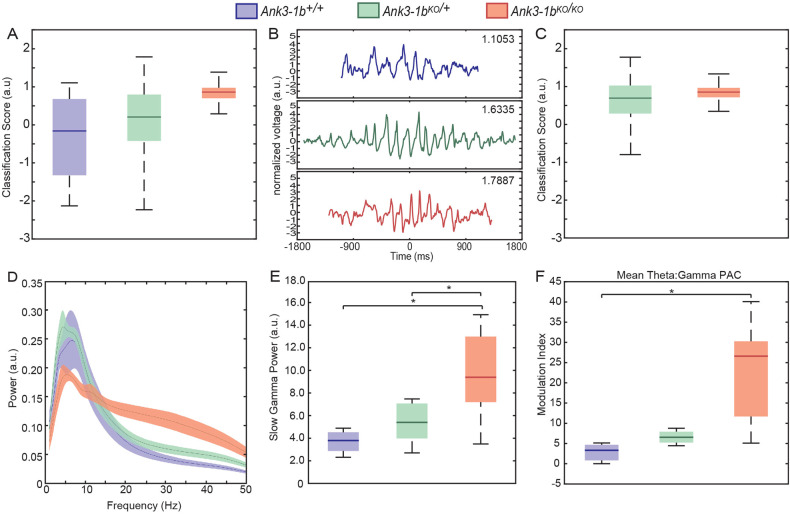
Seizure activity and neocortical rhythms in *Ank3-1b* knockout mice. **A** Boxplots of all positive and negative classification scores of SWD-like activity in EEG recordings from WT and *Ank3-1b*^*KO/+*^ and *Ank3-1b*^*KO/KO*^ mice. **B** Example EEG trace from automated SWD prediction with highest classification score from each genotype. Note: the trace for the WT mouse does not meet the requirements for classification as an SWD, demonstrating the need for **C** manual validation. **C** Boxplots of classification scores after manual verification of SWDs. Note: WT mice had no confirmed SWDs. **D** Average power spectra for all recordings per genotype with 95% confidence intervals. **E** Plots of estimated gamma power (25-45 Hz) for all recordings per genotype with 95% confidence intervals. Significant increases in gamma power are seen between *Ank3-1b*^*KO/KO*^ and WT mice (*p = 0.024) and between *Ank3-1b*^*KO/KO*^ and *Ank3-1b*^*KO/+*^ (*p = 0.015). **F** Average Theta:Gamma PAC (*p = 0.029).

Previous characterization of *Ank3-1b* mice showed that both *Ank3-1b*^*KO/KO*^ and *Ank3-1b*^*KO/+*^ KOs have frequent seizure episodes, with SWDs occupying approximately 4% of total EEG activity from *Ank3-1b*^*KO/KO*^ mice [7]. Since SWD spiking occurs at 6-8 Hz, creating pronounced peaks in power spectra, and because these seizures are accompanied by behavioral arrest, it was important to remove these events from our analyses. To do this, we utilized a supervised learning algorithm to automatically identify seizures in the EEG data [32]. This algorithm identifies SWDs by first identifying consecutive spikes that are at least 2.5 x the average baseline voltage of the overall EEG and then based on the weighed scores of three additional properties: 1.) ~6 Hz spiking frequency (set range from 5 to 10 Hz), 2.) ~16 to 32 Hz harmonic commonly seen in power spectra of SWDs, and 3.) sharpness of spikes as estimated using the D4 wavelet transform. We trained this algorithm on EEG from the *Ank3-1b* model to identify tentative SWDs (Fig. 1A & B) according to scores most resembling a subset of hand-scored data. Though the learning algorithm identified minimal false-positive SWD events (example WT trace seen in Fig. 1B), we chose to manually verify every automated call made by ‘detect_SWD’ (Fig. 1C). This data was then used to index EEGs and remove all SWDs before spectral analysis.

Spectral analysis of all EEG recordings, indiscriminate of behavior, was first conducted to look for large overall differences across genotypes (Fig. 1D). *Ank3-1b* deletion differentially affected the power of the different rhythm types across the entire recording time (Fig. 1D; generalized linear mixed model: significant genotype by rhythm type interaction: F(8,48) = 5.392, *p* < 0.001). The power of delta and slow gamma rhythms, but not theta, significantly differed across genotypes (main effect of genotype on delta power: F(2,16) = 4.085, *p* = 0.037; Fig. 1E, main effect of genotype on gamma power: F(2,16) = 4.038, *p* = 0.038). The slow gamma power that we measured in the ~25–45 Hz range did not appear to be an integer multiple of lower frequency peaks, and the peak was not in the range of typical theta harmonics. This suggests that the gamma that we measured did not merely reflect theta harmonics.

To explore the extent to which slow gamma power was modulated by the phase of lower-frequency rhythms [33], we performed a cross-frequency coupling analysis. Coupling of slow gamma amplitude to the phase of lower frequency rhythms (<10 Hz) was apparent in most of the mice (Supplementary Figs. 2-3). Comparison of theta-gamma phase-amplitude coupling (PAC), but not theta-delta PAC, was stronger in *Ank3-1b*^*KO/KO*^ mice than in wild-type mice (main effect of genotype on theta-delta PAC: F(2,11) = 0.759 p = 0.491; Fig. 1F, the main effect of genotype on theta-gamma PAC: F(2,11) = 6.655, *p* = 0.013; post hoc pairwise comparisons: *Ank3-1b*^*KO/KO*^ vs wildtype theta-gamma PAC: p = 0.029; *Ank3-1b*^*KO/KO*^ vs ; *Ank3-1b*^*KO/+*^ theta-gamma PAC: *p* = 0.051; *Ank3-1b*^*KO/+*^ vs wildtype: *p* = 1). These results support the conclusion that the slow gamma rhythms recorded were physiologically relevant and not simply reflective of noise (EEG traces and gamma filtered traces see Supplementary Fig. 4). We also found a significant relationship between slow gamma power and seizure classification scores (Supplementary Fig. 5).

The presence of these overall differences across genotypes encouraged us to next investigate effects of *Ank3-1b* deletion during specific behavioral and sleep states. We used DeepLabCut (DLC) [34] to obtain estimates of animals’ positions, and a previously published method to classify awake behaviors and sleep [35], from home cage videos (Supplementary Fig. 6).

### *Ank3-1b* KO mice exhibit sleep disturbances co-occurring with increased slow gamma power

**Fig. 2:**
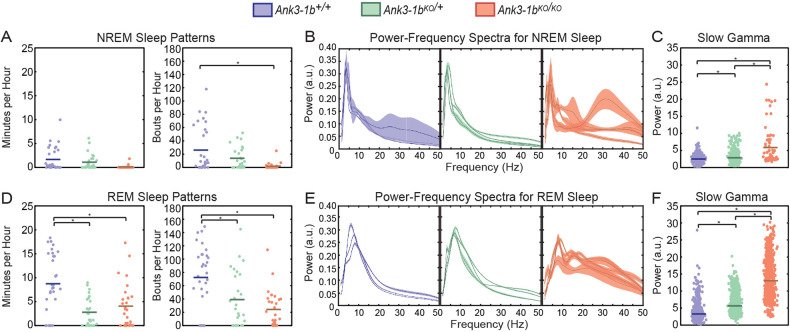
*Ank3-1b* knockout mice showed disturbed sleep patterns and increased slow gamma during sleep, with stronger effects observed during REM sleep than NREM. **A** Plots of time spent in NREM (left) and frequency of NREM bouts (right) per hour in which each point represents an hour of measurement. *Ank3-1b*^*KO/KO*^ mice have increased numbers of NREM bouts compared to WT mice (*p = 0.004). **B** Individual plots of average power spectra during NREM sleep in which each plot represents power measured from an individual mouse with bootstrapped 95% confidence intervals showing variability in power between bouts. **C** Plots of estimated gamma power (25-45 Hz) in which each point is a gamma power measurement during a NREM bout. *Ank3-1b* mice have increased slow gamma power compared to WT during NREM sleep (*Ank3-1b*^*KO/KO*^ vs. WT: *p < 0.001; *Ank3-1b*^*KO/+*^ vs. WT: *p = 0.001; *Ank3-1b*^*KO/KO*^ vs. *Ank3-1b*^*KO/+*^: *p < 0.001). **D** Plots of time spent in REM (left) and frequency of REM bouts (right) per hour in which each point represents an hour of measurement. *Ank3-1b*^*KO/+*^ and *Ank3-1b*^*KO/KO*^ mice have decreased hourly REM sleep duration compared to WT (*p = 0.002 and *p = 0.03, respectively), and *Ank3-1b*^*KO/KO*^ and *Ank3-1b*^*KO/+*^ mice have decreased REM bouts per hour compared to WT (*p = 0.002 and *p < 0.001, respectively). **E** Individual plots of average power spectra during REM sleep in which each plot represents an individual mouse with bootstrapped 95% confidence intervals showing variability in power between bouts**. F** Plots of estimated gamma power (25–45 Hz) in which each point is a gamma power measurement during a REM bout. *Ank3-1b* mice have increased slow gamma compared to WT during REM sleep (*Ank3-1b*^*KO/KO*^ vs. WT: *p < 0.001; *Ank3-1b*^*KO/+*^ vs. WT: *p = 0.02; *Ank3-1b*^*KO/KO*^ vs. *Ank3-1b*^*KO/+*^: *p < 0.001).

Sleep disturbance is a primary symptom of BD present throughout all mood states [36], and sleep disturbance is a common, chronic problem for patients with epilepsy [37]. Thus, we used an existing classifier [35] to analyze sleep patterns in *Ank3-1b* mice. We further used an algorithm that distinguishes REM and NREM in sleep EEG using the theta (6–10 Hz) to delta (2–5 Hz) power ratio [38, 39]. Significant differences in overall sleep duration (i.e., number of minutes of sleep per hour) were not observed between genotypes (Supplementary Fig. 7A). The number of sleep bouts per hour also were not significantly different across genotypes (Supplementary Fig. 7B). However, we found that the effects of *Ank3-1b* deletion on sleep patterns differed across sleep stages, with sleep patterns more strongly affected during REM than non-REM (Fig. 2A, D; Supplementary Fig. 8A, C; significant genotype by sleep stage interaction effects: F(3,168) = 35.7, *p* < 0.001 for time spent in sleep and F(3,169) = 23.1, *p* < 0.001 for number of sleep bouts). *Ank3-1b* deletion did not affect the duration of NREM sleep epochs (Fig. 2A; no significant main effect of genotype on sleep duration: F(2,85) = 1.6, *p* = 0.20), although the number of NREM bouts per hour was significantly lower in *Ank3-1b*^*KO/KO*^ mice than in wild-type mice (significant main effect of genotype: F(2,85) = 5.6, *p* = 0.005; *Ank3-1b*^*KO/KO*^ vs wildtype: t(85) = 3.4, *p* = 0.004; other pairwise comparisons were non-significant). In contrast, the duration of sleep epochs during REM was significantly lower in *Ank3-1b* mice than in wildtype mice (Fig. 2D; main effect of genotype on sleep duration: F(2,84) = 6.8, *p* = 0.002; post-hoc pairwise comparisons: *Ank3-1b*^*KO/KO*^ vs wildtype: t(84) = 2.5, *p* = 0.03; *Ank3-1b*^*KO/+*^ vs wildtype: t(84) = 3.6, *p* = 0.002). The number of REM bouts per hour was also significantly lower in *Ank3-1b* mice than in wild-type mice (Fig. 2D; main effect of genotype on number of sleep bouts: F(2,84) = 13.5, *p* < 0.001; post-hoc pairwise comparisons: *Ank3-1b*^*KO/KO*^ vs wildtype: t(84) = 5.1, *p* < 0.001; *Ank3-1b*^*KO/+*^ vs wildtype: t(84) = 3.4, *p* = 0.002). These findings suggest that *Ank3-1b* deletion more strongly disrupts REM sleep than NREM sleep. It is important to note that due to lack of simultaneous electromyography (EMG) recordings, we were unable to definitively determine if overall REM time and number of REM bouts was reduced, or if this data simply reflects disruption of normal theta-delta ratios characteristic of classic REM sleep. Nonetheless, disrupted REM properties are indicative of many different sleep disorders and are implicated in epilepsy and BD [40–49].

We next examined whether disturbances in REM and NREM sleep patterns were accompanied by disturbances in EEG rhythms during sleep. We found that *Ank3-1b* deletion affected oscillatory power differently during REM and NREM sleep (generalized linear mixed model: significant genotype by sleep stage interaction: F(3,342) = 92.3, *p* < 0.001; significant genotype by sleep stage by rhythm type interaction: F(12,342) = 1178.1, *p* < 0.001), with stronger effects of *Ank3-1b* deletion on power spectra observed during REM than NREM (Fig. 2B, E). Therefore, we next analyzed EEG rhythms during NREM and REM separately.

During identified NREM sleep epochs, *Ank3-1b* deletion did not produce generalized effects on power across all frequencies (generalized linear mixed model: significant genotype by rhythm type interaction: F(6,168) = 53.7, *p* < 0.001). Instead, effects of *Ank3-1b* deletion on rhythmic power were most pronounced for slow gamma rhythms (Fig. 2B, Supplementary Fig. 8B). Slow gamma power was significantly greater in *Ank3-1b* mice than in wildtype mice in a gene-dosage dependent manner (Fig. 2C; main effect of genotype on slow gamma power: F(2,56) = 38.5, *p* < 0.001; post-hoc pairwise comparisons: *Ank3-1b*^*KO/KO*^ vs wildtype slow gamma: t(56) = 8.8, *p* < 0.001; *Ank3-1b*^*KO/+*^ vs wildtype slow gamma: t(56) = 3.5, *p* = 0.001; *Ank3-1b*^*KO/KO*^ vs *Ank3-1b*^*KO/+*^: t(56) = 7.0, *p* < 0.001). *Ank3-1b* deletion also modestly affected delta rhythms, a type of rhythm that is predominant in EEG recordings during NREM [50]. However, in contrast to gamma results, power in the delta frequency range was significantly lower in *Ank3-1b*^*KO/KO*^ mice than in wild-type mice (Supplementary Fig. 9A).

We next investigated how *Ank3-1b* deletion altered EEG rhythms during identified REM sleep epochs (Fig. 2E; Supplementary Fig. 8D; Supplementary Fig. 9B). We again assessed slow gamma rhythms and theta, a rhythm type that dominates EEG recordings during REM sleep [50]. *Ank3-1b* deletion differentially affected the power of the different rhythm types during REM (Fig. 2E; generalized linear mixed model: significant genotype by rhythm type interaction: F(6,174) = 2302.5, *p* < 0.001). The power of slow gamma rhythms during REM significantly differed across genotypes (Fig. 2F, main effect of genotype: F(2,58) = 404.0, *p* < 0.001). Specifically, slow gamma power increased with decreasing *Ank3-1b* copy number (post-hoc pairwise comparisons: wildtype vs *Ank3-1b*^*KO/+*^ slow gamma: t(58) = 2.5, *p* = 0.02*; Ank3-1b*^*KO/+*^ vs *Ank3-1b*^*KO/KO*^ slow gamma: t(58) = 21.6, *p* < 0.001; *Ank3-1b*^*KO/KO*^ vs wildtype slow gamma: t(58) = 27.3, *p* < 0.001). Theta power during REM also significantly differed across genotypes (main effect of genotype: F(2,58) = 94.8, *p* < 0.001). However, in contrast to slow gamma results, homozygous *Ank3-1b* deletion significantly decreased theta power during REM (Supplementary Fig. 9B). Also, theta power during REM did not significantly differ between wildtype mice and *Ank3-1b*^*KO/+*^ mice. Taken together, this collection of results suggests a potential relationship between increased slow gamma power and disrupted REM sleep.

Previous characterization of these mice showed that *Ank3-1b*^*KO/KO*^ mice have premature mortality rates [7], and meet the criteria for sudden death in epilepsy (SUDEP), so we also looked at seizure activity during REM and NREM sleep. Our data indicates that seizures are approximately 10X more common during REM (1.24 seizures/hr) than during NREM (0.13 seizures/hr) sleep, and this increased seizure frequency was statistically significant in REM compared to NREM sleep (Supplementary Fig. 10A, B). This result was striking because seizures triggered during REM sleep are more likely to cause sudden death compared to waking and NREM sleep [51]. We then looked at the effect of seizure occurrence on slow gamma power in *Ank3-1b*^*KO/+*^ and *Ank3-1b*^*KO/KO*^ mice and found that seizure occurrence affected gamma power differently between genotypes during REM sleep. We found that seizure occurrence had no significant effect on gamma power in *Ank3-1b*^*KO/+*^, but seizure occurrence was associated with significantly increased gamma power in *Ank3-1b*^*KO/KO*^ mice (Supplementary Fig. 10C). This highlights again a positive correlation between seizure phenotype severity and increased slow gamma power and suggests that seizure activity and slow gamma rhythms may be associated. When we looked at the effect of seizure occurrence on theta power in *Ank3-1b*^*KO/+*^ and *Ank3-1b*^*KO/KO*^ mice during REM sleep, we found that seizure occurrence affected theta power differently between genotypes. We found that seizure occurrence had no significant effect on theta power in *Ank3-1b*^*KO/+*^, but seizure occurrence was associated with significantly decreased theta power in *Ank3-1b*^*KO/KO*^ mice (Supplementary Fig. 10C). Seizures were extremely rare during NREM; only a single mouse exhibited seizures during NREM. Thus, we did not examine gamma power for seizures occurring during NREM.

Again, it is important to note that seizures may be unaccounted for due to potentially unidentified sleep bouts, since thorough analysis of sleep requires simultaneous EMG analysis. Mice may be having seizures during REM and NREM periods that our algorithm was unable to identify due to disrupted theta-to-delta ratios. Thus, it is critical for future studies to further explore these effects in order to better understand the risk and underlying mechanisms leading to SWDs during sleep in this model.

### *Ank3-1b* KO mice exhibit hyperactivity during awake behaviors

**Fig. 3:**
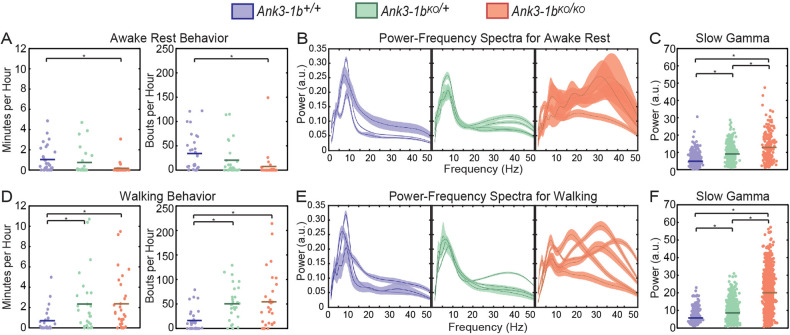
*Ank3-1b* knockout mice exhibited hyperactivity and increased slow gamma power during awake behaviors. **A** Plots of time spent in awake rest per hour (left) and bouts of awake rest per hour (right) in which each point represents an hour of measurement. *Ank3-1b*^*KO/KO*^ mice spent decreased time per hour in awake rest (*p = 0.006) and had decreased bouts of awake rest (*p = 0.04) compared to WT mice. **B** Individual plots of average power spectra during awake rest in which each plot represents an individual mouse with bootstrapped 95% confidence intervals showing variability in power between bouts. **C** Plots of estimated gamma power (25–45 Hz) in which each point is a gamma power measurement during a bout of awake rest. *Ank3-1b* mice have increased slow gamma compared to WT mice during awake rest (*p < 0.001, for all comparisons). **D** Plots of time spent walking per hour (left) and bouts of walking per hour (right) in which each point represents an hour of measurement. *Ank3-1b*^*KO/KO*^ and *Ank3-1b*^*KO/+*^ mice spent increased time (*p < 0.001 and *p = 0.04, respectively) and had increased bouts of walking per hour (*p = 0.002 and *p < 0.001, respectively) compared to WT mice. **E** Individual plots of average power spectra during walking in which each plot represents an individual mouse with bootstrapped 95% confidence intervals showing variability in power between bouts. **F** Plots of estimated gamma power (25-45 Hz) in which each point is a gamma power measurement during a bout of walking. *Ank3-1b* mice had increased slow gamma power compared to WT mice during walking (*p < 0.001, for all comparisons).

Since activity levels are altered in depressive and manic states of BD and can similarly be affected during preictal, interictal, and postictal phases of epilepsy [5, 52], we looked at awake rest and walking behaviors in *Ank3-1b* mice (Fig. 3A, D; Supplementary Fig. 11A, C). The number of awake rest and walking bouts per hour were differentially affected by *Ank3-1b* deletion (Fig. 3A, D; significant genotype by behavior type interaction: F(2,168) = 4.8, *p* = 0.001). Compared to wild-type mice, *Ank3-1b*^*KO/KO*^ mice exhibited significantly fewer bouts of awake rest per hour (Fig. 3A; generalized linear mixed model, the significant main effect of genotype: F(2,84) = 5.4, *p* = 0.006; post-hoc pairwise comparisons: wildtype vs *Ank3-1b*^*KO/+*^: t(84) = 2.2, *p* = 0.07; *Ank3-1b*^*KO/KO*^ vs *Ank3-1b*^*KO/+*^: t(84) = 0.9, *p* = 0.4; *Ank3-1b*^*KO/KO*^ vs wildtype: t(84) = 3.2, *p* = 0.006) and spent less time (minutes per hour) in the awake rest state (Fig. 3A; significant main effect of genotype: F(2,84) = 3.9, *p* = 0.002; post-hoc pairwise comparisons: wildtype vs *Ank3-1b*^*KO/+*^: t(84) = 2.1, *p* = 0.7; *Ank3-1b*^*KO/KO*^ vs *Ank3-1b*^*KO/+*^: t(84) = 0.2, *p* = 0.9; *Ank3-1b*^*KO/KO*^ vs wildtype: t(84) = 2.6, *p* = 0.04). Regarding walking behavior, significantly more walking bouts per hour were observed for *Ank3-1b* mice compared to wild-type mice (Fig. 3D; significant main effect of genotype: F(2,84) = 12.8, *p* < 0.001; post-hoc pairwise comparisons: wildtype vs *Ank3-1b*^*KO/+*^: t(84) = 4.9, *p* < 0.001; *Ank3-1b*^*KO/KO*^ vs wildtype: t(84) = 3.4, *p* = 0.002). Also, *Ank3-1b* mice spent significantly more time walking compared to wildtype mice (Fig. 3D; significant main effect of genotype: F(2,84) = 8.6, *p* < 0.001; post hoc pairwise comparisons: *Ank3-1b*^*KO/KO*^ vs wildtype: t(84) = 3.2, *p* = 0.04; *Ank3-1b*^*KO/+*^ vs wildtype: t(84) = 4.1, *p* < 0.001). These data support the claim for a manic-like phenotype in *Ank3-1b* mice previously reported [31].

### *Ank3-1b* KO mice exhibit increased slow gamma power during awake rest and walking states

Delta rhythms are often evident during periods of awake rest in rodents [53–55], and low-frequency theta can be associated with non-moving states [56]. Therefore, we included measurements of these rhythm types, together with slow gamma rhythms, in our model when testing for differences in the power of EEG rhythms between genotypes during awake rest (Fig. 3B, C; Supplementary Fig. 11B). During awake rest, the different rhythm types were differentially affected by *Ank3-1b* deletion, with largest effects observed for slow gamma rhythms (Fig. 3B; generalized linear mixed model, significant genotype by rhythm type interaction effect: (F(6,159) = 173.8, *p* < 0.001). Slow gamma power increased as *Ank3-1b* levels decreased across the three genotypes (Fig. 3C; significant main effect of genotype: F(2,53) = 94.0, *p* < 0.001). *Ank3-1b*^*KO/KO*^ and *Ank3-1b*^*KO/+*^ mice had significantly increased slow gamma power compared to wildtype mice, and *Ank3-1b*^*KO/KO*^ mice had significantly higher slow gamma than *Ank3-1b*^*KO/+*^ mice (post-hoc pairwise comparisons: wildtype vs. *Ank3-1b*^*KO/+*^: t(53) = 8.4, *p* < 0.001; *Ank3-1b*^*KO/KO*^ vs *Ank3-1b*^*KO/+*^: t(53) = 7.2, *p* < 0.001; *Ank3-1b*^*KO/KO*^ vs wildtype: t(53) = 12.8, *p* < 0.001). In contrast, power in the theta frequency range was not significantly affected by *Ank3-1b* deletion (Supplementary Fig. 12A), and delta power was lower in *Ank3-1b*^*KO/KO*^ mice than in wild-type mice (Supplementary Fig. 12B).

We next assessed the effects of *Ank3-1b* deletion on EEG rhythms during walking behavior. Active walking behavior is associated with prominent theta and gamma activity in local field potential recordings from rodents [57–59]. Thus, during walking, we tested for theta and slow gamma power differences across genotypes (Fig. 3E, F; Supplementary Fig. 10D; Supplementary Fig. 12C). We found that theta and slow gamma rhythms during walking were differentially affected by *Ank3-1b* deletion (generalized linear mixed model, significant genotype by rhythm type interaction effect: F(3,112) = 75.5, *p* < 0.001). Slow gamma rhythms during walking were significantly increased by *Ank3-1b* deletion (Fig. 3F; significant main effect of genotype on slow gamma: F(2,56) = 189.3, *p* < 0.001). As was observed during awake rest, slow gamma power increased with decreasing *Ank3-1b* copy number (post hoc pairwise comparisons: wildtype vs. *Ank3-1b*^*KO/+*^: t(56) = 4.9, *p* < 0.001; *Ank3-1b*^*KO/KO*^ vs *Ank3-1b*^*KO/+*^: t(56) = 13.2, *p* < 0.001; *Ank3-1b*^*KO/KO*^ vs wildtype: t(56) = 18.9, *p* < 0.001). In contrast, theta power during walking was significantly decreased in *Ank3-1b* mice compared to wildtype mice (Supplementary Fig. 12C). It is interesting to note that peak slow gamma power was greater than theta power in several of the *Ank3-1b*^*KO/KO*^ mice (see Fig. 3E), lending further support to the interpretation that slow gamma power increases were not due to theta harmonics.

### *Ank3-1b* KO mice exhibit increases in repetitive behaviors

**Fig. 4:**
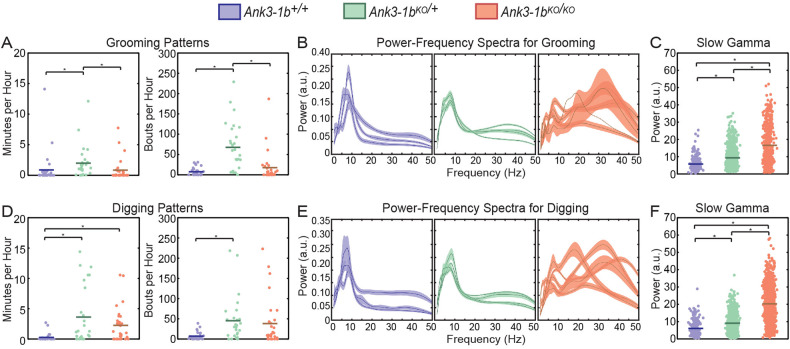
*Ank3-1b* knockout mice exhibited increased repetitive behaviors and increased slow gamma power during repetitive behaviors. **A** Plots of grooming time (left) and grooming bouts (right) per hour in which each point represents an hour of measurement. *Ank3-1b*^*KO/+*^ mice had increased repetitive grooming bouts compared to *Ank3-1b*^*KO/KO*^ and WT mice (*p < 0.001, both), and spent more time grooming per hour compared to *Ank3-1b*^*KO/KO*^ and WT mice (*p < 0.001, both). **B** Individual plots of average power spectra during grooming in which each plot represents an individual mouse with bootstrapped 95% confidence intervals showing variability of power between bouts. Note that one *Ank3-1b*^*KO/KO*^ mouse only had one bout of grooming, so no confidence intervals are shown. **C** Plots of estimated gamma power (25–45 Hz) in which each point is a gamma power measurement during a bout of grooming. *Ank3-1b* mice have increased slow gamma power compared to WT during grooming (*p < 0.001, for all comparisons). **D** Plots of digging time (left) and digging bouts (right) per hour in which each point represents an hour of measurement. *Ank3-1b*^*KO/+*^ and *Ank3-1b*^*KO/KO*^ mice spent more time digging per hour compared to WT mice (*p = 0.02, both), and *Ank3-1b*^*KO/+*^ mice had more digging bouts per hour compared to WT mice (*p = 0.002). **E** Individual plots of average power spectra during digging in which each plot represents an individual mouse with bootstrapped 95% confidence intervals showing variability between bouts. **F** Plots of estimated gamma power (25–45 Hz) in which each point is a gamma power measurement during a bout of digging. *Ank3-1b* mice had increased slow gamma power compared to WT mice during digging (*p < 0.001, for all comparisons).

Another common endophenotype of BD and epilepsy is obsessive and compulsive thoughts and behaviors [60–62]. One way to test for such characteristics in mice is to look at their propensity for repetitive behaviors, such as repetitive grooming and digging. Repetitive behaviors are another characteristic related to BD that has not previously been explored in *Ank3-1b* mice. Thus, we compared the frequency and duration of grooming and digging behaviors in *Ank3-1b* KO and wildtype mice (Fig. 4A, D; Supplementary Fig. 13A, C). We found a significant effect of *Ank3-1b* deletion on repetitive grooming bouts and amount of time spent grooming per hour (Fig. 4A; generalized linear mixed model, the main effect on genotype: bouts: F(2,84) = 36.3, *p* < 0.001; time: F(2,84) = 44.5, *p* < 0.001). However, only heterozygous but not homozygous *Ank3-1b* deletion significantly altered grooming bouts (post-hoc pairwise comparisons: wildtype vs *Ank3-1b*^*KO/+*^: t(84) = 7.4, *p* < 0.001; *Ank3-1b*^*KO/KO*^ vs *Ank3-1b*^*KO/+*^: t(84) = 7.6, *p* < 0.001; *Ank3-1b*^*KO/KO*^ vs. wildtype: t(84) = 0.05, *p* = 1.0). Also, only *Ank3-1b*^*KO/+*^ but not *Ank3-1b*^*KO/KO*^ mice showed significantly increased time grooming (generalized linear mixed model, main effect on genotype: wildtype vs. *Ank3-1b*^*KO/+*^: t(84) = 8.7, *p* < 0.001; *Ank3-1b*^*KO/KO*^ vs *Ank3-1b*^*KO/+*^: t(84) = 8.1, *p* < 0.001; *Ank3-1b*^*KO/KO*^ vs. wildtype: t(84) = 0.7, *p* = 0.5). Digging may provide a better measure of repetitive behaviors in *Ank3-1b* mice because, unlike grooming, it does not require animals to balance on their hind paws, which may be more difficult for *Ank3-1b*^*KO/KO*^ mice due to their mild ataxia [32]. A significant effect of *Ank3-1b* deletion on digging behaviors was observed (Fig. 4D) when digging was measured in bouts per hour (generalized linear mixed model, main effect of genotype: F(2,84) = 6.6, *p* = 0.002). As was the case with grooming, only *Ank3-1b*^*KO/+*^ mice and not *Ank3-1b*^*KO/KO*^ mice showed significantly more digging bouts per hour compared to wildtype mice (post-hoc pairwise comparisons: wildtype vs *Ank3-1b*^*KO/+*^: t(84) = 3.6, *p* = 0.002; *Ank3-1b*^*KO/KO*^ vs. *Ank3-1b*^*KO/+*^: t(84) = 2.5, *p* = 0.03; *Ank3-1b*^*KO/KO*^ vs wildtype: t(84) = 1.3, *p* = 0.21). We also found a significant effect of *Ank3-1b* deletion on minutes per hour of repetitive digging (generalized linear mixed model, main effect on genotype: F(2,84) = 5.5, p = 0.006). Unlike with grooming, both *Ank3-1b*^*KO/+*^ mice and *Ank3-1b*^*KO/KO*^ mice showed significantly increased digging time compared to wildtype mice (generalized linear mixed model, main effect on genotype: wildtype vs *Ank3-1b*^*KO/+*^: t(84) = 2.8, p = 0.02; *Ank3-1b*^*KO/KO*^ vs *Ank3-1b*^*KO/+*^: t(84) = 0.2, p = 0.9; *Ank3-1b*^*KO/KO*^ vs wildtype: t(84) = 2.9, p = 0.02). It is interesting to note that while one would expect the magnitude of behavioral effects to increase with reduced *Ank3-1b* dosage, *Ank3-1b*^*KO/+*^ mice exhibited the strongest repetitive behaviors phenotype. This may be due to the confounding factor of ataxia in *Ank3-1b*^*KO/KO*^ mice [63], making it difficult for *Ank3-1b*^*KO/KO*^ mice to engage in prolonged bouts of coordinated grooming and digging movements.

### *Ank3-1b* KO mice show increased slow gamma rhythms during repetitive behaviors

Power spectra during grooming and digging behaviors showed peaks in the theta and slow gamma bands, so we next examined whether *Ank3-1b* deletion affected theta and slow gamma power during grooming and digging (Fig. 4B-C, E-F; Supplementary Fig. 13B, D; Supplementary Fig. 14). As was observed for other awake behaviors, different effects of *Ank3-1b* deletion on theta and slow gamma power were observed during grooming behaviors (Fig. 4B, generalized linear mixed models, significant genotype by rhythm type interaction effect on grooming behaviors: F(3,136) = 114.8, *p* < 0.001). Specifically, slow gamma power was increased by *Ank3-1b* deletion in a dosage-dependent manner (Fig. 4C, generalized linear mixed model, significant main effect of genotype: F(2,60) = 59.6, *p* < 0.001; post-hoc pairwise comparisons: wildtype vs *Ank3-1b*^*KO/+*^: t(60) = 3.8, *p* < 0.001; *Ank3-1b*^*KO/+*^ vs *Ank3-1b*^*KO/KO*^: t(60) = 7.6, *p* < 0.001; *Ank3-1b*^*KO/KO*^ vs wildtype: t(60) = 10.8, *p* < 0.001). In contrast, *Ank3-1b* deletion decreased theta power during grooming (Supplementary Fig. 14A). Similar to slow gamma power during grooming, slow gamma power during digging was increased by *Ank3-1b* deletion in a dosage-dependent manner (Fig. 4F; generalized linear mixed model, significant main effect of genotype: F(2,68) = 118.2, *p* < 0.001; post-hoc pairwise comparisons: wildtype vs *Ank3-1b*^*KO/+*^: t(68) = 5.8, *p* < 0.001; *Ank3-1b*^*KO/+*^ vs *Ank3-1b*^*KO/KO*^: t(68) = 10.0, *p* < 0.001; *Ank3-1b*^*KO/KO*^ vs wildtype: t(68) = 15.4, *p* < 0.001) while theta power was unchanged (Supplementary Fig. 14B). Indeed, analyses revealed that *Ank3-1b* deletion affected slow gamma power similarly during the different types of repetitive behaviors (i.e., grooming and digging; generalized linear mixed model, no significant main effect of repetitive behavior type on slow gamma power: F(1,128) = 0.41, *p* = 0.525; no significant genotype by repetitive behavior type interaction effect: F(1, 128) = 0.27, *p* = 0.76). Therefore, we included slow gamma measurements from grooming and digging behaviors together and again assessed the effect of *Ank3-1b* deletion on slow gamma rhythm power. As was observed for grooming and digging behaviors alone, *Ank3-1b* deletion increased slow gamma power during grooming and digging behaviors analyzed together (generalized linear mixed model, main effect of genotype: F(2,128) = 91.9, *p* < 0.001). During grooming and digging behaviors, slow gamma power in both *Ank3-1b*^*KO/+*^ and *Ank3-1b*^*KO/KO*^ mice was larger than slow gamma power in wildtype mice (post-hoc pairwise comparisons: wildtype vs *Ank3-1b*^*KO/+*^: t(128) = 3.9, *p* < 0.001; *Ank3-1b*^*KO/KO*^ vs wildtype: t(128) = 13.2, *p* < 0.001). Also, slow gamma power during grooming and digging behaviors was greater in *Ank3-1b*^*KO/KO*^ mice than in *Ank3-1b*^*KO/+*^ mice (*Ank3-1b*^*KO/+*^ vs *Ank3-1b*^*KO/KO*^: t(128) = 9.7, *p* < 0.001). Taken together, these findings suggest that increased slow gamma rhythms accompany increased repetitive behaviors in the *Ank3-1b* mice.

## Discussion

Human genetic studies have advanced our understanding of psychiatric illness through the identification of rare and common variants associated with complex neurodevelopmental disorders. However, understanding the role that such variants play in polygenic traits like mood disturbances seen in BD and epilepsy has remained a challenge. One such challenge is the difficulty of modeling subjective human traits like mood in rodents. Even more difficult is modeling rhythmic disorders like BD and epilepsy in which shifts in mood and activity levels occur on various time scales as long as weeks to months.

Many studies attempting to utilize *Ank3* as a model of BD in mice have shown promise. For example, one study showed that *Ank3-1b*^*KO/+*^ mice model aspects of BD such as shifts from manic-like features, including reduced anxiety and increased motivation for reward, to depressive-like features after chronic stress that were attenuated by lithium [31]. However, these behaviors were of small effect size and difficult to reproduce in small-scale studies. Alternatively, a pyramidal cell forebrain conditional KO of *Ank3* exhibited similar behavioral phenotypes modeling BD but with greater effect size [64]. In this model, behaviors at baseline were manic-like, including hyperactivity, and changed to depressive-like features after social defeat stress. This phenotype was also attenuated by lithium and valproate. However, the genetic manipulation was more severe than the diminished *ANK3* expression reported in human studies, and neither study modeled the full range of BD symptoms and endophenotypes. Furthermore, the seizure phenotype was unknown at the time of these studies, so attention was not given to the relevance of these behaviors to epilepsy.

These limitations illustrate the trade-off that exists between models with BD-related phenotypes of large effect size and those that more accurately model BD variable expressivity but have phenotypes of smaller effect size. This trade-off affects overall reproducibility in rodent experiments and their translational relevance to BD treatment in human patients. Our approach was able to identify new BD-related behaviors in *Ank3-1b* mice and provides advanced methodology that can be tailored towards more reliable long-term behavioral analysis of mice. We developed algorithms suitable for analyzing long periods of undisturbed home cage activity that more faithfully capture behavioral rhythmicity seen in BD and epilepsy.

Furthermore, our results illustrate a robust behavioral and electrophysiological phenotype that can be used for future in vivo single-unit electrophysiological experiments conducted over periods of weeks to months with the intention of gaining mechanistic insights into behaviors relevant to mood symptoms seen in human BD and epilepsy patients. For example, the sleep disturbance phenotype that we have identified in this model has direct relevance to patient treatment of BD and epilepsy because sleep disturbances are present across all states of both disorders and highly consequential for treatment [65]. While manic states of BD are characterized by decreased need for sleep and depressive states of BD are characterized by increased sleep, even euthymic states of BD show characteristics of sleep disturbance [40–47]. Sleep disturbance is common among patients with epilepsy, and seizures during sleep, particularly during REM sleep, can be fatal [51, 66].

Recently, a quantitative phosphoproteomic study has implicated *Ank3* in sleep need, lending support to our findings of dysregulated sleep in *Ank3-1b* mice [67]. Future studies utilizing EMG will be helpful for studying specific types of REM disturbances in *Ank3-1b* mice and further understanding the role of *Ank3-1b* in sleep need. Interestingly, there is growing evidence for a role of REM sleep in emotional memory processing in the hippocampus [68–71], and gamma is thought to promote memory consolidation during REM sleep [72]. Additionally, our data is consistent with phenotypes seen in the Kv3.1/Kv3.3 and Stargazer mouse models. Like *Ank3*, Kv3-type channels are expressed in interneurons, and Kv3.1 is expressed only in PV interneurons. Deletion of Kv3.1/Kv3.3 in PV interneurons leads to decreased action potential dynamic range, hyperactivity, and increased slow gamma power (20-60 Hz) [73–76]. The Stargazer mouse model also has many overlapping features with the *Ank3-1b* mouse model. For example, both models exhibit the absence epilepsy and ataxia, respond to ethosuximide, have PV-interneuron dysfunction, and show an increase in interictal slow gamma power (Maheshwari et al., 2015). A recent study characterized the behaviors of the Stargazer model during home-cage activity and found overlapping phenotypes with *Ank3-1b* mice, including hyperactivity, increased repetitive behaviors, and sleep deficits (Schirmer et al., 2022).

Given previous studies showed reduced PV interneuron firing rates in ranges above 100 Hz in *Ank3-1b*^*KO/KO*^ mice [7], we hypothesize that the increased slow gamma power seen here was due to an inability of PV interneurons in KO mice to fire at frequencies high enough to produce fast gamma oscillations. Unfortunately, we were unable to measure fast gamma in this study due to the low sampling frequency of the pre-existing dataset. PV interneurons in the cortex and hippocampus have been shown to be more active during REM than NREM [77]; the PV interneuron dysfunction in *Ank3-1b* mice could explain the larger effect of *Ank3-1b* loss on REM sleep compared to NREM sleep, and why seizures are more common during REM sleep, in this model. Furthermore, we hypothesize that the increased slow gamma seen during other behavioral states may relate to changes in behavior that we were unable to identify here due to limitations of the existing dataset, including limited recording durations, sample size, and camera angles for distinguishing more complex behaviors.

Further studies examining the relationship between the increases in slow gamma power observed here, and changes in power of other frequency bands, with changes in *Ank3-1b* mice behavior will be important for understanding the human relevance of these mice as a model of BD. For example, theta-gamma coupling is highly implicated in processes of memory and navigation in both humans and rodents [78], and gamma coherence is disrupted during various tasks in human subjects with BD [27, 79]. Studies of gamma in BD have shown increased power in the 30-50 Hz range in BD patients during visual tasks and resting states [28, 29, 80]. Additionally, the global increase we see in slow gamma across behaviors may reflect volume-conducted signals from specific brain regions involved in different behaviors, so in vivo recordings using implanted probes will be critical for identifying the sources of these signals. This increased slow gamma phenotype in *Ank3-1b* mice may represent another way in which EEG disturbances can be used in translational studies to develop treatments for patients with BD.

### Supplementary information

Supplementary Information

## Data Availability

The datasets generated and analyzed during the current study are available from the corresponding author on reasonable request.

## Code availability

The codes used for data analysis are freely accessible from the GitHub repository (https://github.com/jevillacres/DLC-Code) for research purposes.
