# Supplementary material for: Abnormal patterns of sleep and waking behaviors are accompanied by neocortical oscillation disturbances in an Ank3 mouse model of epilepsy-bipolar disorder comorbidity
Source: Transl Psychiatry. 2023 Dec 20;13:403. doi: 10.1038/s41398-023-02700-2 (PMC10733341; doi:10.1038/s41398-023-02700-2)
Supplement: Supplementary file 1 — Supplementary Information [file 41398_2023_2700_MOESM1_ESM.docx]

**Supplementary Information for**

**Abnormal patterns of sleep and waking behaviors are accompanied by neocortical oscillation disturbances in an *Ank3* mouse model of epilepsy-bipolar disorder comorbidity**

Juan E Villacres^1,2,3Ɨ^, Nicholas Riveira^1,2,4Ɨ^, Sohmee Kim^1,2^, Laura L Colgin^1,2,5^, Jeffrey L Noebels^6,7,8^, and Angel Y Lopez^1,2^*

**The PDF file includes:**

- Materials and Methods
- References
- Supplementary Table T1
- Supplementary Figures S1-S14

**MATERIALS AND METHODS**

**Animals**

All studies here are secondary analyses of existing paired video-EEG recordings previously published^1^. This dataset utilized adult (6-8 weeks old) C57BL/6J *Ank3-1b* mice bred from heterozygous crosses to generate test mice and littermate controls (Supplementary Table 1). Both sexes were included in our analyses. This dataset consisted of 4 wildtype (WT), 7 *Ank3-1b^KO/+^*, and 7 *Ank3-1b^KO/+^* mice. However, only 3 WT, 4 *Ank3-1b^KO/+^*, and 6 *Ank3-1b^KO/+^* mice had accompanying video data. Therefore, data from this subset were used for behavior-related analyses, while all 18 mice were examined in the general EEG recording spectral analyses indiscriminate of behavioral state. One *Ank3-1b^KO/KO^* mouse was excluded from spectral analysis due to excessive seizure activity (average of ~665 seizures/hour representing 43.7% of the recording time). All mice were housed on a reverse light-dark cycle, and the animal procedures followed protocols approved by the IACUC committees of Baylor College of Medicine.

**Video-electroencephalography**

Video-EEG recording methods were described in detail previously^1^. Mice were surgically implanted with bilateral silver wire electrodes (0.005^″^ diameter) inserted through cranial burr holes into the subdural space over the frontal and parietal cortices bilaterally. The electrodes were attached to a micro-miniature connector cemented to the skull, and simultaneous video-EEG recordings (Harmonie software version 6.1c, Stellate Systems) were obtained from freely moving mice in a home cage setup. Video was collected at 30 frames per second, and EEG was sampled at 200-250 Hz. This low EEG sampling rate precluded rigorous analysis of signals in higher frequency ranges (e.g., >80 Hz) due to the Engineer’s Nyquist Rule. Thus, we limited our spectral analyses to signals in the 1-50 Hz range to ensure at least 4 sampling points for any recorded oscillation cycle. The home cage setup consisted of a glass tank (LWH: 12.25” x 6.25” x 8.25”) with an open lid, filled with ~1 inch of corn cob bedding and freely accessible food pellets and water. The cages were housed in facilities with restricted human access to minimize stress and interference. Behavior was monitored by video recording from a single camera angle, either perpendicular to the long side or short end of the cage.

**Movement tracking and behavioral classification**

We used DeepLabCut (DLC), an open-source marker-less pose estimation software^2^, to track the position of freely moving mice in video recordings. Since video angles were not consistent across all mice and some videos contained multiple cages of singly housed mice, multiple models were trained to ensure high accuracy position tracking for all mice. In total, 7 different models were trained on various cage positions and video cropping parameters. From each set of videos with similar angles, 200-250 randomly selected frames were labeled, and its model trained through DeepLabCut using resources from the Texas Advanced Computing Center for 300,000+ iterations.

The position estimates gathered from DLC were used to build two types of classifiers. One type of classifier utilized linear discriminant analysis (LDA) models to classify awake behaviors. The other was a binary classifier based on previously published work^3^ and was used to identify sleep episodes. Two hours of manually scored behaviors (i.e. walking, grooming, digging, awake rest, and sleep) from two mice per genotype were used as ground truth for training and validating each model.

For the sleep classifier, DLC data tracking each mouse’s nose, front paws, hind paws, base of tail, and mid-body were used to evaluate the position centroids of each frame. Frame-by-frame centroid displacements were then evaluated to track each mouse’s motion over time. Displacement percentiles were then calculated for motion thresholding. The classifier’s receiver operating characteristic (ROC) curve was generated using the manually scored data as ground truth to select the displacement threshold that provided optimal true positive and false positive rates, which was found to be the 85th percentile (Supplementary Figure 6A). A bout duration threshold of 40 seconds was also used as previously described^3^. All bouts in which the centroid displacement was lower than the 85th displacement percentile for at least 40 seconds were thus classified as sleeping bouts.

Awake rest and walking behaviors were classified using LDA on the kinematic information from the videos taken for bouts lasting longer than 0.5 seconds. Awake rest states were defined as periods during which the mouse was not moving but was not identified as asleep by the sleep classifier. 140 features for building the LDA classifiers were generated from the DLC position data. The features consisted of the Euclidean distance, angles, and displacement vectors between each point. Manually scored data from each behavior was established as ground truth for training and cross validation. LDA models were built using MATLAB’s fitcdiscr function. K-Fold cross-validation with 500 partitions followed by generation of ROC curves was performed for each classifier to evaluate their performance (Supplementary Figure 6B-E). The classifiers were subsequently used to identify grooming, digging, walking, and resting awake behaviors.

**Signal processing and spectral analysis**

We used a supervised learning algorithm, ‘detect_SWDs’^4^, to identify seizure events from EEG recordings for removal prior to spectral analyses. Seizure activity was defined by at least 3 consecutive spike and wave discharges (SWDs) with amplitudes greater than or equal to 2.5 × baseline voltages at frequencies of 5–10 Hz. A ‘detect_SWDs’ classifier was trained on one-hour EEG recordings from two *Ank3-1b^KO/KO^* mice, and subsequently used to identify seizures in all mice. The accuracy of the trained classifier was 91.3%. SWD calls for all mice were visually verified using a ‘detect_SWDs’ GUI and cross-validated with previously hand-scored data to eliminate all false negative and false positive calls from the data (Figure 1A-C). This algorithm allowed for indexing of validated SWDs from the raw EEG before spectral analyses.

Signals from the left frontal lobe (AP: ~2 mm, ML: ~1.5 mm), where the electrode furthest from the digitally averaged reference electrode was located, were used in all spectral analyses. Signals were first normalized to a variance of 1 and a mean of 0 using code from the spike wave detection toolbox^4^ and low pass filtered at 50 Hz. Normalized power of the EEG signal was then estimated from signal frequencies 1 - 50 Hz in 0.2 Hz wide frequency bands. A Morlet wavelet transform of the EEG signal was used for estimating oscillatory power across frequencies as previously described^5,6^, with a width parameter (σ) of 5 and a frequency resolution of 1 Hz. Average power spectra are presented with corresponding 95% bootstrapped confidence intervals that were created by randomly sampling power spectra from recordings during individual bouts of behavior.

For non-behavior-specific analyses of full EEG recordings, all interictal clips longer than 4 seconds were analyzed using the Morlet wavelet transform method described above. To obtain a single estimate of average slow gamma rhythm power, the mean power estimates across 25-45 Hz frequency bins were summed.

For phase-amplitude coupling (PAC), the ictal portions for the EEG of each mouse were removed prior to analysis using timestamps recorded from the spike-wave detector, and the remaining EEG was concatenated to form one continuous interictal EEG signal (Supplementary Figures 2 & 3). The sampling frequency was selected appropriately as 200 Hz for those mice with only EEG recordings and 250 Hz for those mice with EEG and electrocardiograph recordings. The recordings were then split into individual signals 4000 sample points in length for processing efficiency, with each signal being treated as a sample for PAC analysis. PAC analysis was conducted using a toolbox previously described by Onslow and colleagues^7^. The PAC of the EEG signal was then displayed using the modulation index as the PAC metric, with phase-frequency and amplitude-frequency vectors encompassing frequencies across delta, theta, and gamma frequency ranges. The standard phase-amplitude coupling for each mouse was then normalized to the maximum value of each mouse.

**Statistics**

All statistics were performed in SPSS (IBM, version 27.0). Data were analyzed using the generalized linear mixed model (GLMM) function. Genotype was included in each model as a fixed effect. Individual mice were subjects, with repeated measurements collected from each mouse. For analysis of average oscillatory power during entire recordings (Figure 1E), repeated measures included hour and rhythm type (i.e., delta, theta, and slow gamma). For sleep patterns analyses (Figure 2A, D, Supplementary Figure 7), repeated measures included hour (i.e., time of day when recording was collected) and sleep stage (i.e., REM vs NREM). A genotype by sleep stage interaction effect was also included in the model. For analysis of oscillatory power during sleep (Figure 2C, F), repeated measures included hour, sleep stage, and rhythm type. Genotype by sleep stage, genotype by rhythm type, and 3-way genotype by sleep stage by rhythm type interaction effects were also included in the model to test whether genotype differentially affected rhythms of different types and whether effects differed across sleep stages. For analysis of genotype differences on awake behaviors (Figures 3A, D; 4A, D), repeated measures included hour and type of behavior. A genotype by behavior type interaction effect was also included in the model. When significant interaction effects were observed, analyses were performed separately for different behavior types, with hour as a repeated measure and genotype tested as a main effect. For analyses of oscillatory power during awake behaviors (Figure 3C, F; 4C, F), repeated measures included hour and rhythm type. Also, a genotype by rhythm type interaction effect was included. When significant interaction effects were found, analyses were performed separately for different rhythms, with hour as a repeated measure and genotype tested as a main effect. Specific details for each comparison are provided in the results. When main effects of genotype were observed, all possible pairwise comparisons of genotypes were performed and adjusted for multiple comparisons using the sequential Bonferroni procedure.

**REFERENCES**

1 Lopez, A. Y., Wang, X., Xu, M., Maheshwari, A., Curry, D., Lam, S. *et al.* Ankyrin-G isoform imbalance and interneuronopathy link epilepsy and bipolar disorder. *Mol Psychiatry* **22**, 1464-1472, doi:10.1038/mp.2016.233 (2017).

2 Mathis, A., Mamidanna, P., Cury, K. M., Abe, T., Murthy, V. N., Mathis, M. W. *et al.* DeepLabCut: markerless pose estimation of user-defined body parts with deep learning. *Nat Neurosci* **21**, 1281-1289, doi:10.1038/s41593-018-0209-y (2018).

3 Singh, S., Bermudez-Contreras, E., Nazari, M., Sutherland, R. J. & Mohajerani, M. H. Low-cost solution for rodent home-cage behaviour monitoring. *PLoS One* **14**, e0220751, doi:10.1371/journal.pone.0220751 (2019).

4 Pfammatter, J. A., Maganti, R. K. & Jones, M. V. An automated, machine learning-based detection algorithm for spike-wave discharges (SWDs) in a mouse model of absence epilepsy. *Epilepsia Open* **4**, 110-122, doi:10.1002/epi4.12303 (2019).

5 Tallon-Baudry, C., Bertrand, O., Delpuech, C. & Pernier, J. Oscillatory γ-Band (30–70 Hz) Activity Induced by a Visual Search Task in Humans. *The Journal of Neuroscience* **17**, 722-734, doi:10.1523/jneurosci.17-02-00722.1997 (1997).

6 Colgin, L. L., Denninger, T., Fyhn, M., Hafting, T., Bonnevie, T., Jensen, O. *et al.* Frequency of gamma oscillations routes flow of information in the hippocampus. *Nature* **462**, 353-357, doi:10.1038/nature08573 (2009).

7 Onslow, A. C. E., Bogacz, R. & Jones, M. W. Quantifying phase–amplitude coupling in neuronal network oscillations. *Progress in Biophysics and Molecular Biology* **105**, 49-57, doi:https://doi.org/10.1016/j.pbiomolbio.2010.09.007 (2011).

**SUPPLEMENTARY FIGURES**


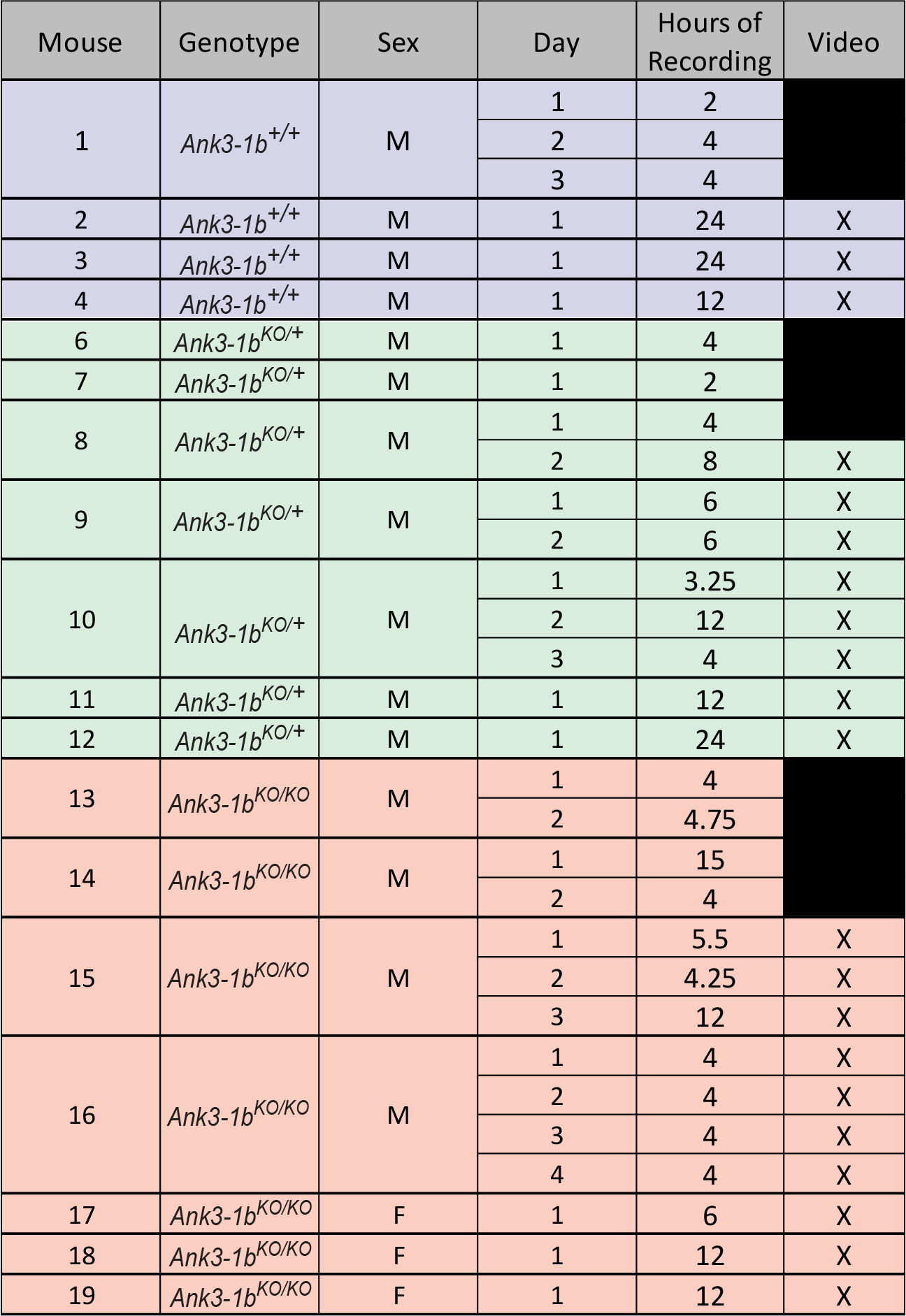


**Supplementary Table 1—Summary of mouse data.** Information regarding the genotype and sex of the mice used for these analyses are included, and the number of days, hours of recording, and which mice had paired video recordings available (X) are specified.


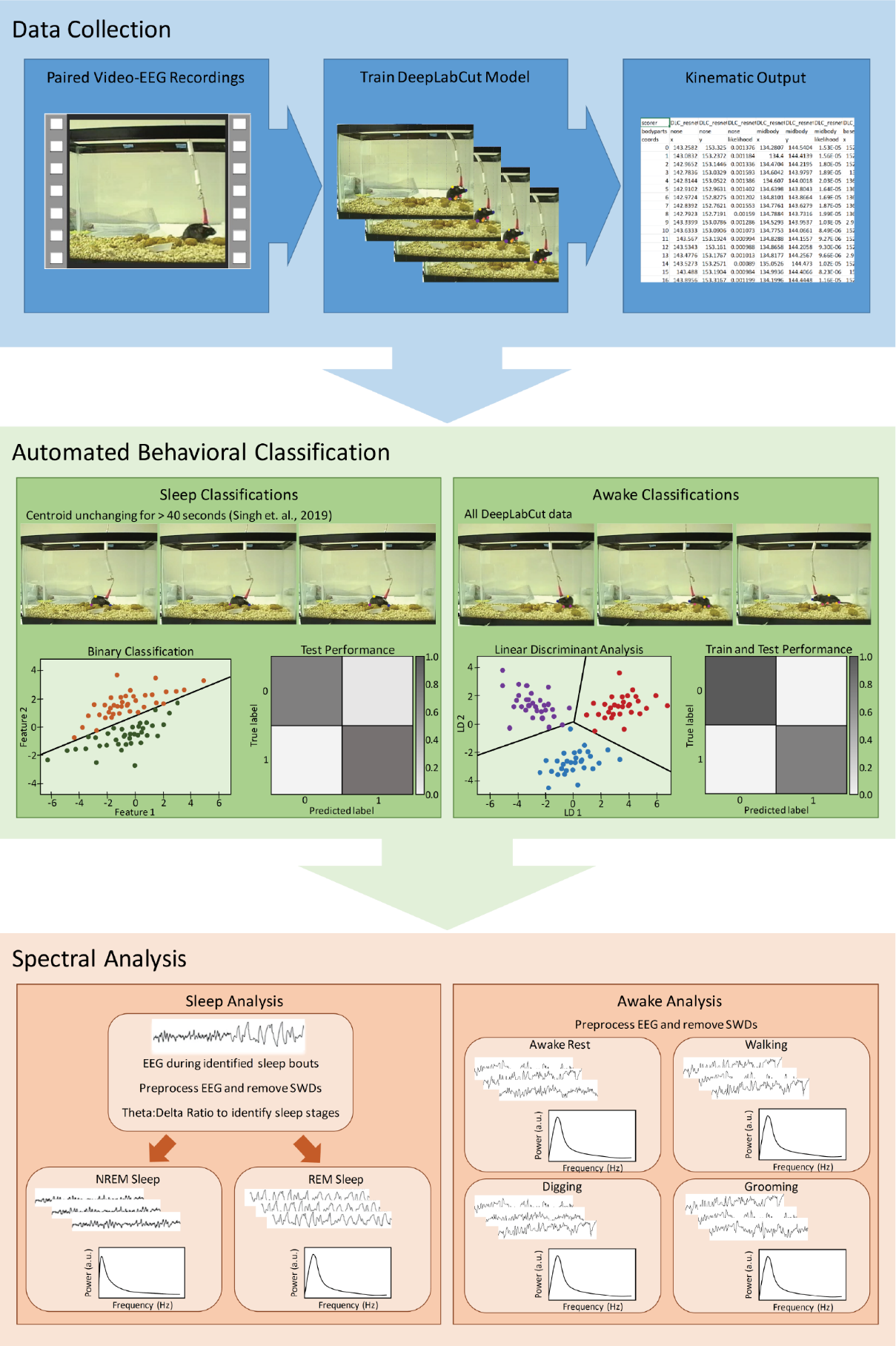


**Supplementary Figure 1—Summary of methodological workflow.** Paired video-EEG recordings were taken overnight, and DeepLabCut (DLC) was trained over 200-250 randomly selected frames to track the animal’s movement from the video recordings taken at 30 frames per second. The kinematic output from DLC was then used to train classifiers to identify sleep and awake behaviors. A binary classifier was used to identify sleep bouts, and linear discriminant analysis was used to develop classifiers for awake rest, walking, digging, and grooming behaviors. EEG during these behaviors was then isolated, seizures were removed, and spectral analysis was conducted. The theta:delta ratio was used to distinguish REM from NREM sleep from EEG during identified sleep bouts.


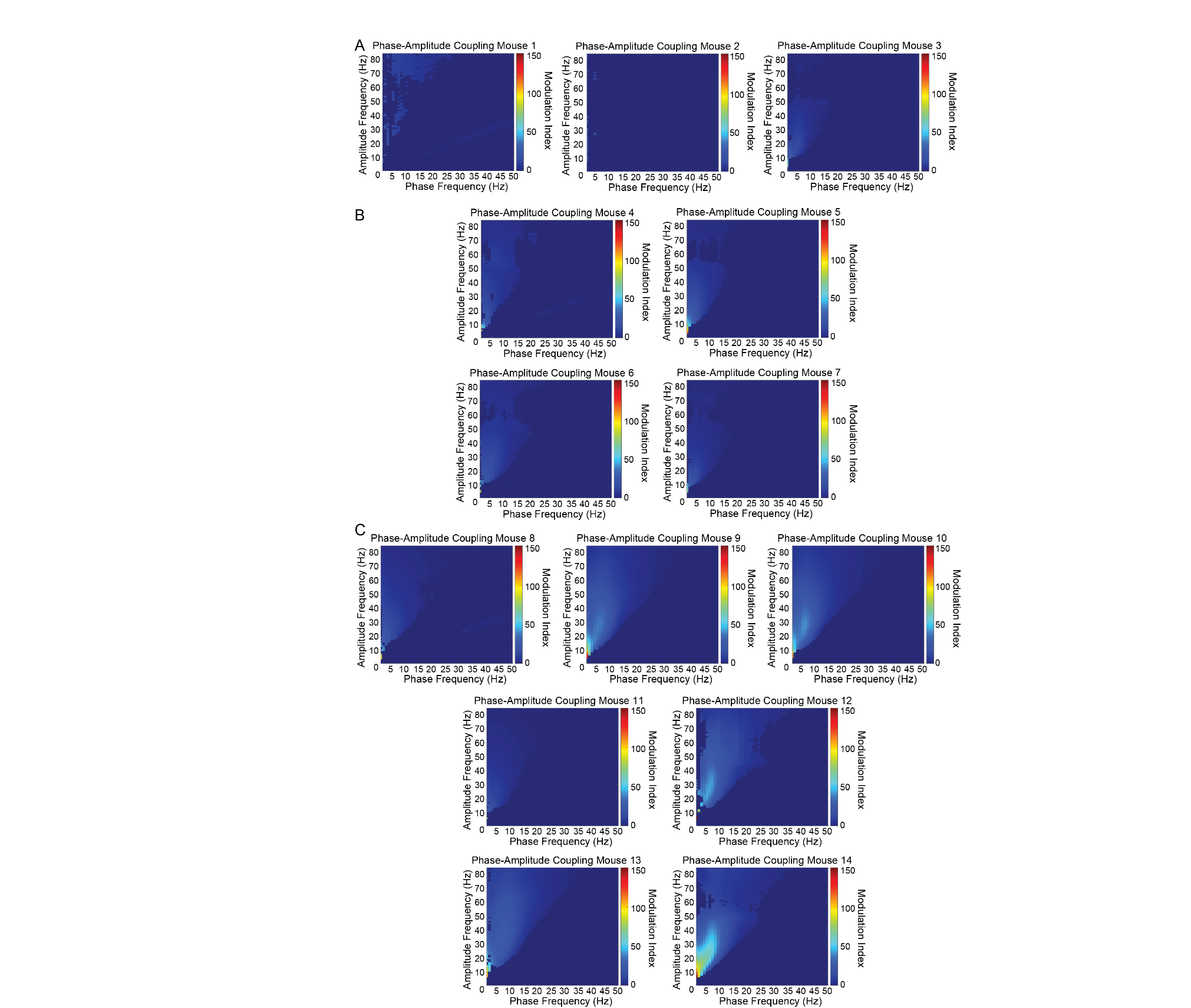


**Supplementary Figure 2—Normalized Phase-Amplitude Coupling (PAC).** Plots were normalized to show relative modulation indices between animals. **A:** Plots showing normalized PAC for individual WT mice. **B:** Plots showing normalized PAC for individual *Ank3-1b^KO/+^* mice. **C:** Plots showing normalized PAC for individual *Ank3-1b^KO/KO^* mice.


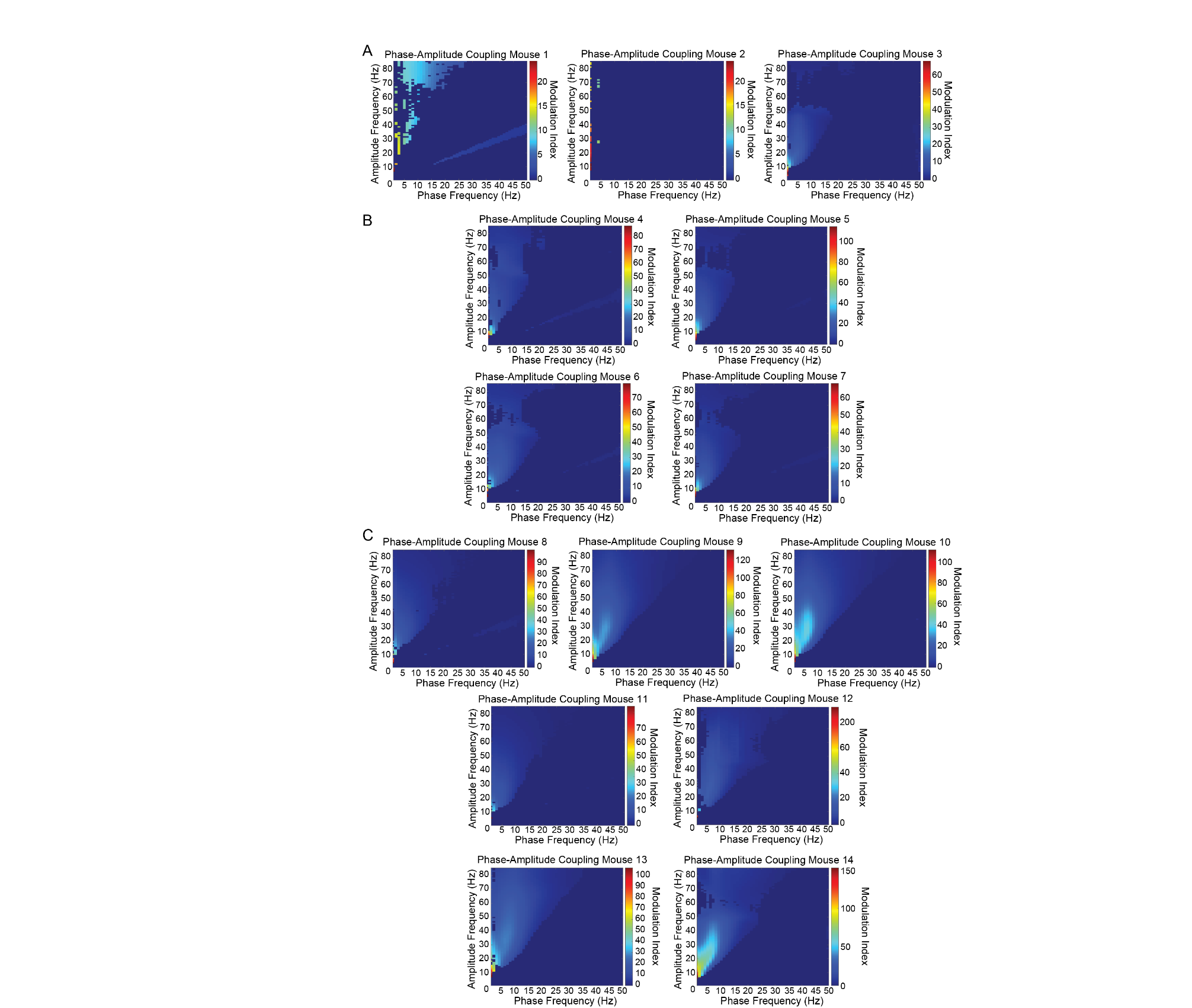


**Supplementary Figure 3—Raw Phase-Amplitude Coupling (PAC).** Plots of raw PAC data to show differences in modulation indices between frequencies within individual animals. **A:** Plots showing PAC for individual WT animals. **B:** Plots showing normalized PAC for individual *Ank3-1b^KO/+^* mice. **C:** Plots showing normalized PAC for individual *Ank3-1b^KO/KO^* mice.


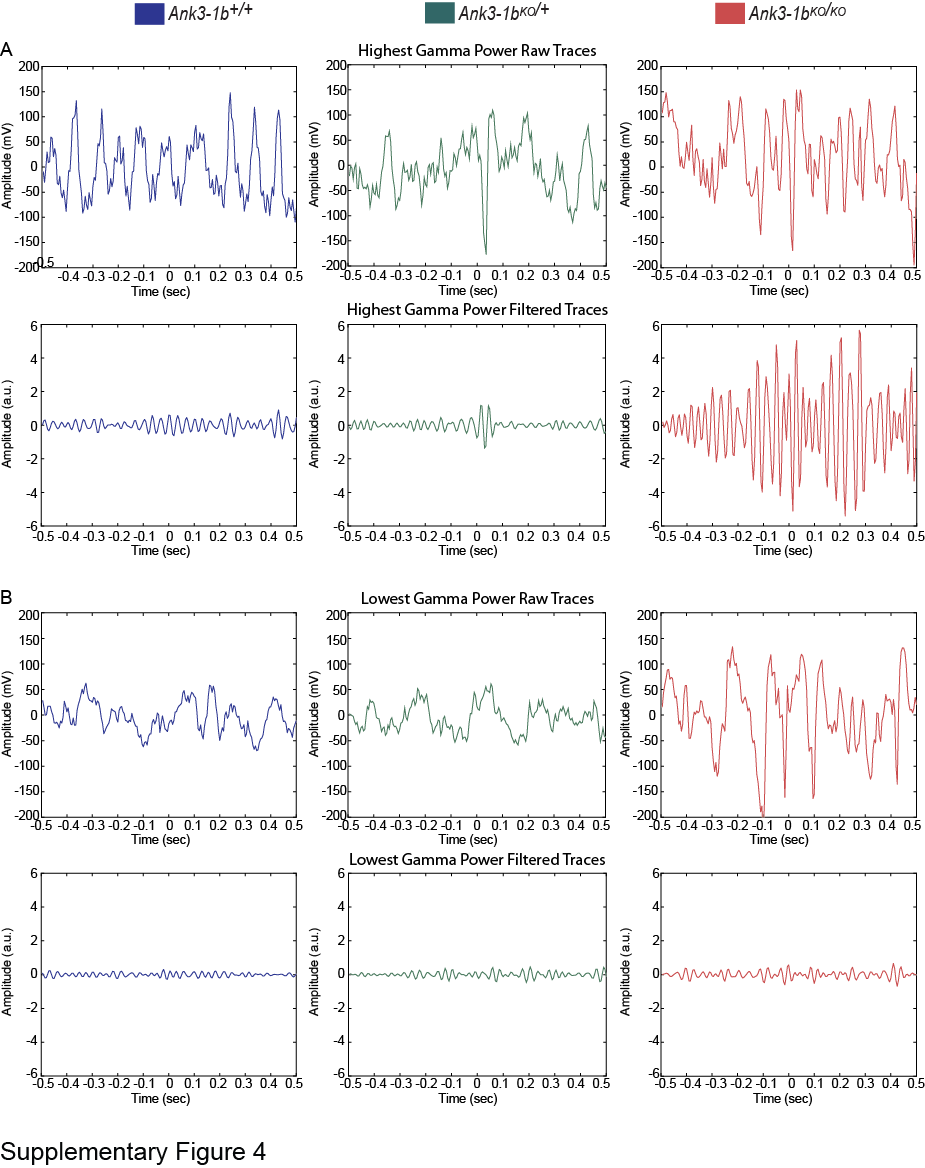


**Supplementary Figure 4—Raw and gamma filtered EEG traces showing episodes with strong and weak gamma power. A:** Raw EEG traces with strong slow gamma power (> 2 std above the mean slow gamma power) from the three genotypes (top row), and their corresponding gamma filtered traces (bottom row). **B:** Raw EEG traces with weak slow gamma power (< 2 std below the mean slow gamma power) from the three genotypes (top row), and their corresponding gamma filtered traces (bottom row).

**
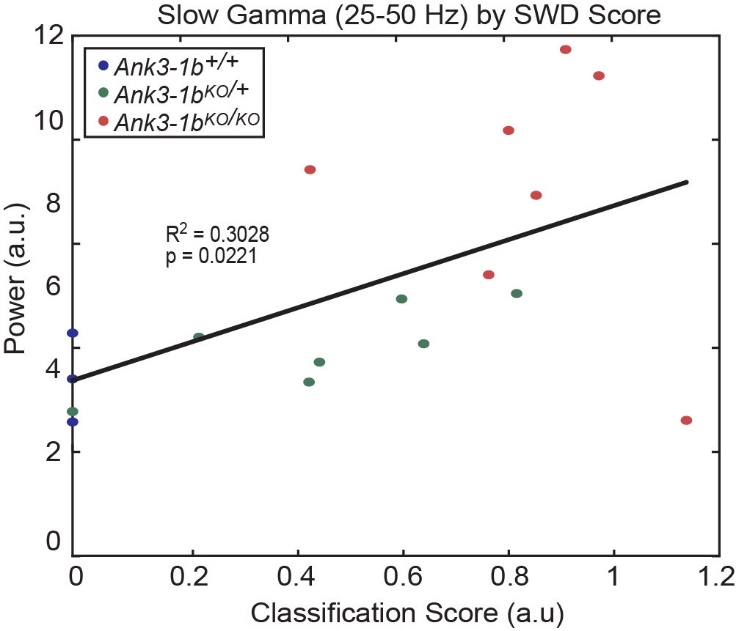
**

**Supplementary Figure 5—Correlation of slow gamma power and SWD classification score.** SWD scores and normalized gamma power values representing the mean across all recordings per mouse are plotted and show a positive correlation between slow gamma power and SWD classification scores (R^2^ = 0.30; *p = 0.02).


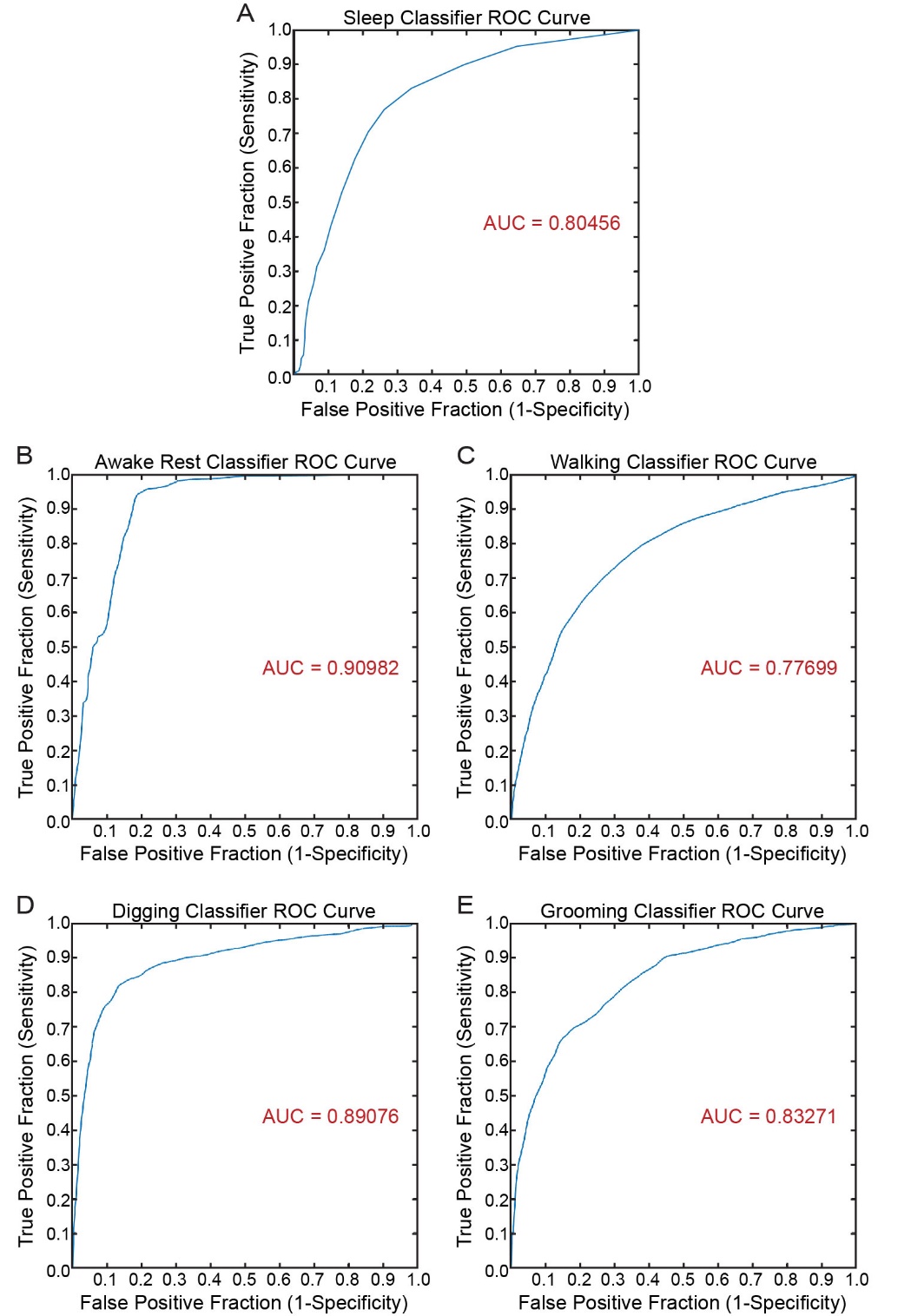


**Supplementary Figure 6—Receiver operator characteristic curves for classifiers. A:** Receiver operator characteristic (ROC) curve for sleep classifier. Area under the curve is 0.80456. The selected threshold corresponds to a true positive fraction of 0.625358 and false positive fraction of 0.175047. **B:** ROC curve for awake rest. Area under the curve is 0.90982. **C:** ROC curve for walking classifier. Area under the curve is 0.77699. **D:** ROC curve for digging classifier. Area under the curve is 0.89076. E: ROC curve for grooming classifier. Area under the curve is 0.83271.

**
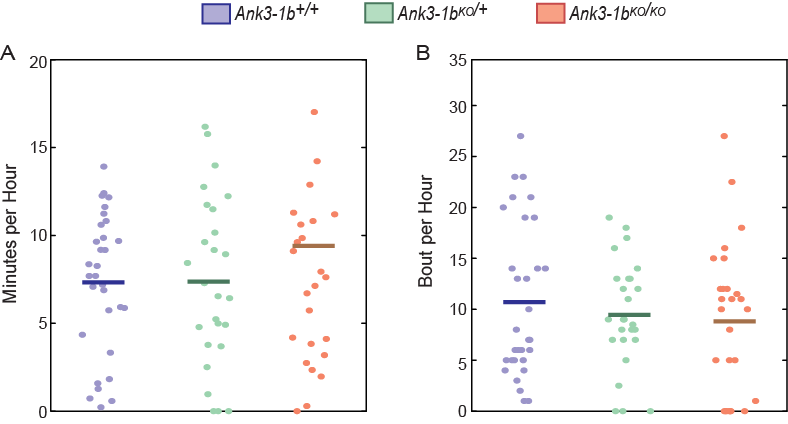
**

**Supplementary Figure 7—Overall sleep time and number of sleep bouts in *Ank3-1b* knockout mice. A:** Plots of total sleep time per hour in which each point represents an hour of measurement (generalized linear mixed model, main effect of genotype: F(2,81) = 1.4, p = 0.3). **B:** Plots of total sleep bouts per hour in which each point represents an hour of measurement (generalized linear mixed model, main effect of genotype: F(2,81) = 0.5, p = 0.6).


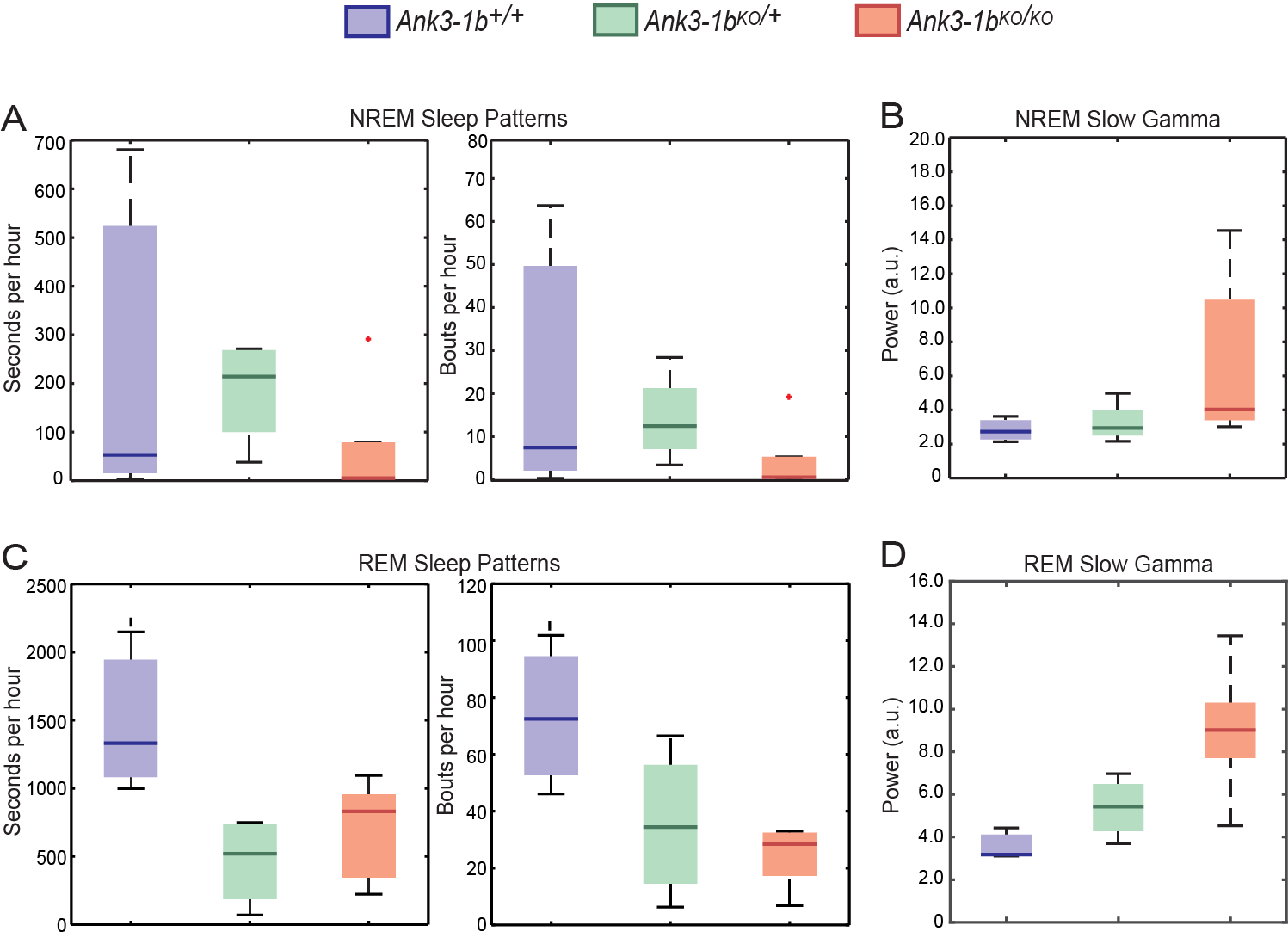


**Supplementary Figure 8—Distribution of individual mouse sleep data. A:** Boxplots showing the distribution of average estimated slow gamma power (25-45 Hz) for mice per genotype during NREM sleep (WT: n = 3 mice; *Ank3-1b^KO/+^*: n = 4 mice; *Ank3-1b^KO/KO^*: n = 4 mice). Note that one *Ank3-1b^KO/KO^* mouse could not be used for spectral analysis because its EEG recordings during NREM bouts were too short after seizure removal. **B:** Boxplots showing the distribution of average time spent in NREM (left) and average frequency of NREM bouts (right) per hour (WT: n = 3 mice; *Ank3-1b^KO/+^*: n = 4 mice; *Ank3-1b^KO/KO^*: n = 5 mice). **C:** Boxplots of average estimated slow gamma power (25-45 Hz) for mice per genotype during REM sleep (WT: n = 3 mice; *Ank3-1b^KO/+^*: n= 4 mice; *Ank3-1b^KO/KO^*: n = 5 mice). **D:** Boxplots showing the distribution of average time spent in REM (left) and average frequency of REM bouts (right) per hour (WT: n = 3 mice; *Ank3-1b^KO/+^*: n = 4 mice; *Ank3-1b^KO/KO^*: n = 5 mice).


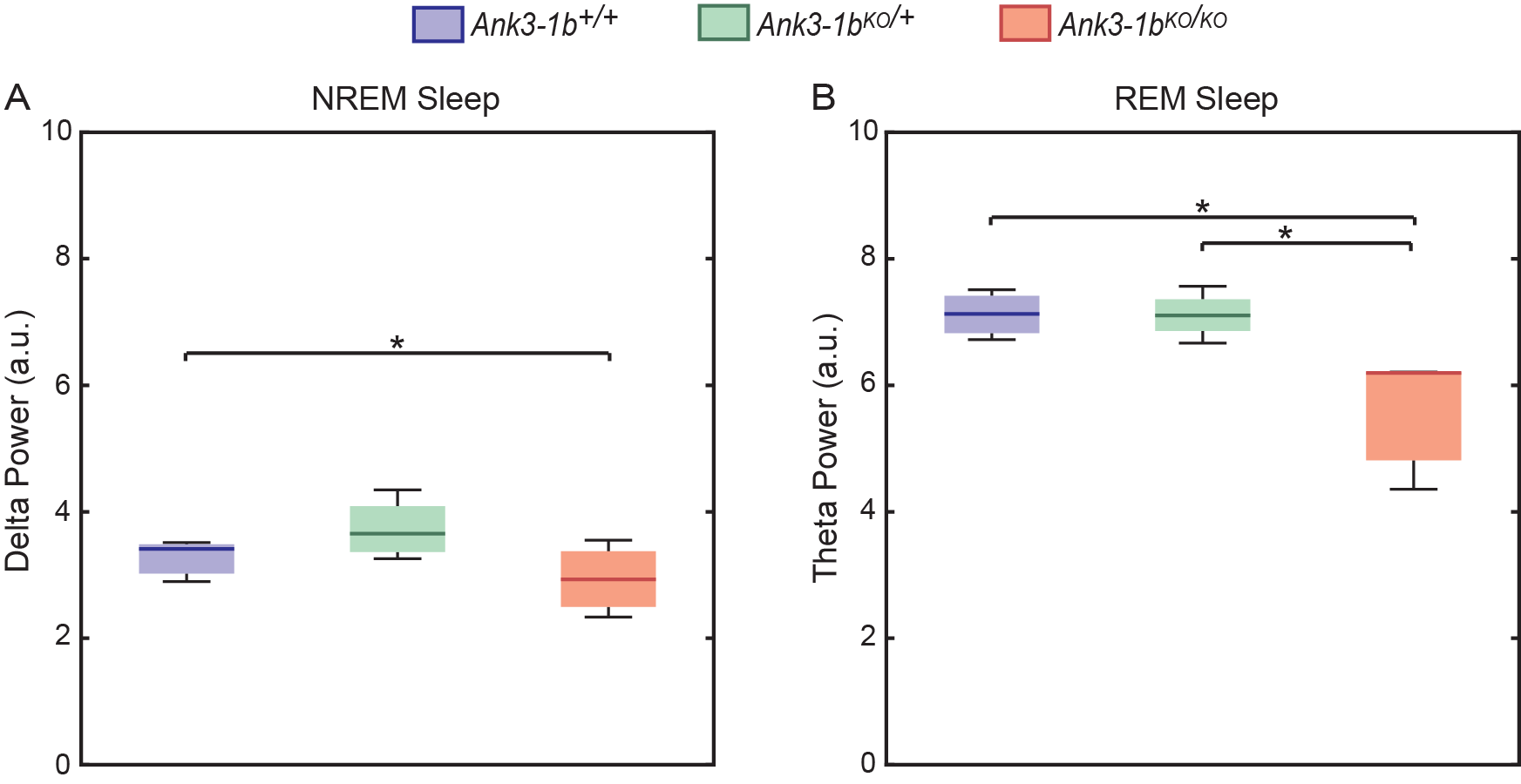


**Supplementary Figure 9—*Ank3-1b^KO/KO^* mice have reduced delta and theta power during sleep. A:** Boxplots of estimated delta power (2-5 Hz) during NREM sleep showing that *Ank3-1b^KO/KO^* mice have decreased delta power compared to WT (post-hoc pairwise comparisons: *Ank3-1b^KO/KO^* vs wildtype theta: t(58) = 6.2, *p < 0.001; wildtype vs *Ank3-1b^KO/+^* theta: t(58) = 1.9, p = 0.07). **B:** Boxplots of estimated theta power (6-10 Hz) during REM sleep showing that *Ank3-1b^KO/KO^* mice have decreased theta power compared to WT and *Ank3-1^KO/+^* ( post-hoc pairwise comparisons: *Ank3-1b^KO/KO^* vs wildtype delta: t(56) = 2.4, *p = 0.04; *Ank3-1b^KO/+^* vs wildtype delta: t(56) = 1.0, p = 0.34; *Ank3-1b^KO/KO^* vs *Ank3-1b^KO/KO^*: t(56) = 3.3, *p = 0.005).


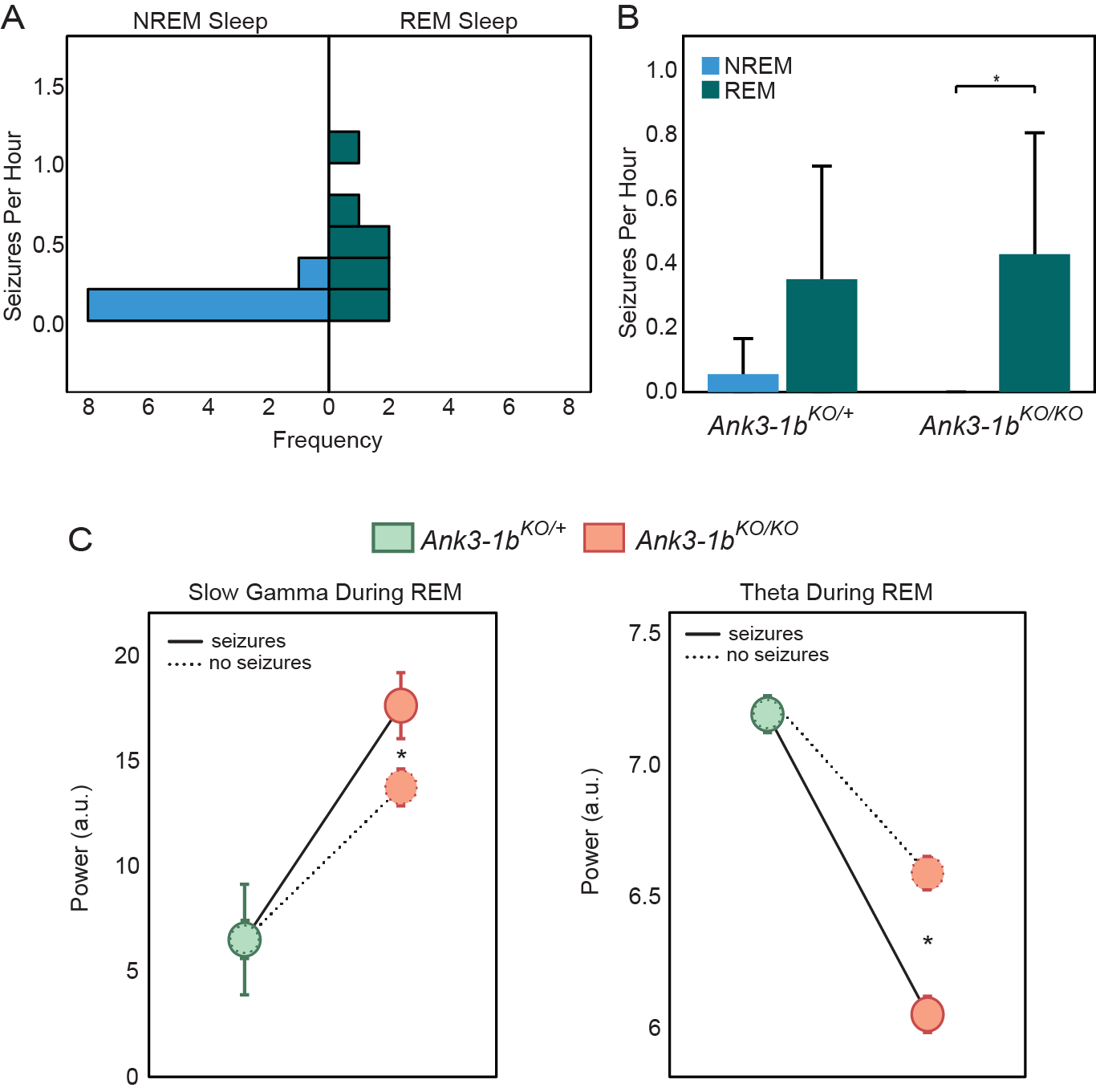


**Supplementary Figure 10—Seizure frequencies differ in REM and NREM sleep and show distinct changes in gamma and theta power. A:** Histogram of seizure frequency during REM (green) and NREM (blue), showing that SWDs are more common during REM than during NREM sleep. **B:** Plots of average seizure frequencies in *Ank3-1b^KO/+^* and *Ank3-1b^KO/KO^* showing that *Ank3-1b^KO/KO^* mice have more seizures during REM sleep (green; n = 9, mean rank = 12.11) than NREM sleep (blue; n = 9, mean rank = 6.89; *p = 0.040, std error = ±9.957). **C:** Comparison of power in gamma and theta frequency bands during REM sleep bouts that did not have seizure activity (dotted lines) compared to those that did have seizure activity (solid lines, left; generalized linear mixed model: significant genotype by seizure occurrence interaction: F(2,38) = 15.3, p < 0.001). During REM, seizure occurrence had no significant effect on gamma power in *Ank3-1b^KO/+^* (seizure occurrence vs. no seizure occurrence: F(1,19) = 0.5; t(19) = 0.7, p = 0.5), but seizure occurrence was associated with significantly increased gamma power in *Ank3-1b^KO/KO^* mice (seizure occurrence vs. no seizure occurrence: F(1,19) = 404; t(19) = 20.1, *p < 0.001). REM bouts during which SWDs in *Ank3-1b^KO/KO^* occurred exhibited greater decreases in theta power compared to bouts during which SWDs did not occur (*p < 0.001, right). During NREM, seizure occurrence affected theta power differently between genotypes (right; significant genotype by seizure occurrence interaction: F(2,38) = 114.7, *p < 0.001). Seizure occurrence had no significant effect on theta power in *Ank3-1b^KO/+^* (seizure occurrence vs. no seizure occurrence: F(1,19) = 4.1; t(19) = 2.0, p = 0.06), but seizure occurrence was associated with significantly decreased theta power in *Ank3-1b^KO/KO^* mice (seizure occurrence vs. no seizure occurrence: F(1,19) = 228.5; t(19) = 15.1, *p < 0.001).


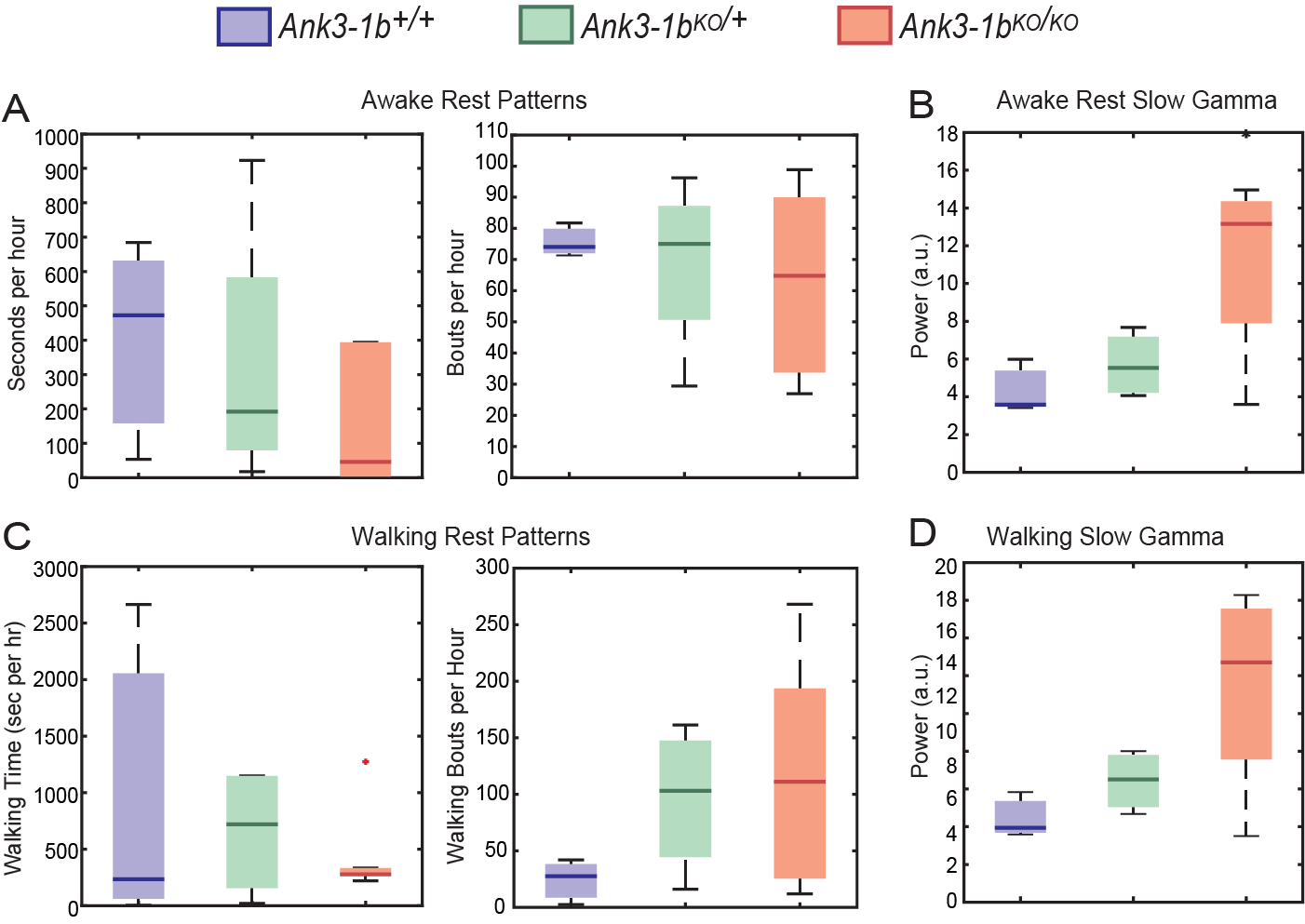


**Supplementary Figure 11—Distribution of individual mouse waking data. A:** Boxplots showing the distribution of average time spent in awake rest (left) and average frequency of awake rest bouts (right) per (WT: n = 3 mice; *Ank3-1b^KO/+^*: n = 4 mice; *Ank3-1b^KO/KO^*: n = 5 mice **B:** Boxplots showing the distribution of average estimated slow gamma power (25-45 Hz) for mice per genotype during awake rest (WT: n = 3 mice; *Ank3-1b^KO/+^*: n = 4 mice; *Ank3-1b^KO/KO^*: n = 4 mice). Note that one *Ank3-1b^KO/KO^* mouse could not be used for spectral analysis because it was seizing during all awake rest bouts.). **C:** Boxplots showing the distribution of average time spent walking (left) and average frequency of walking bouts (right) per hour (WT: n = 3 mice; *Ank3-1b^KO/+^*: n = 4 mice; *Ank3-1b^KO/KO^*: n = 5 mice). **D:** Boxplots of average estimated slow gamma power (25-45 Hz) for mice per genotype during walking (WT: n = 3 mice; *Ank3-1b^KO/+^*: n= 4 mice; *Ank3-1b^KO/KO^*: n = 5 mice).


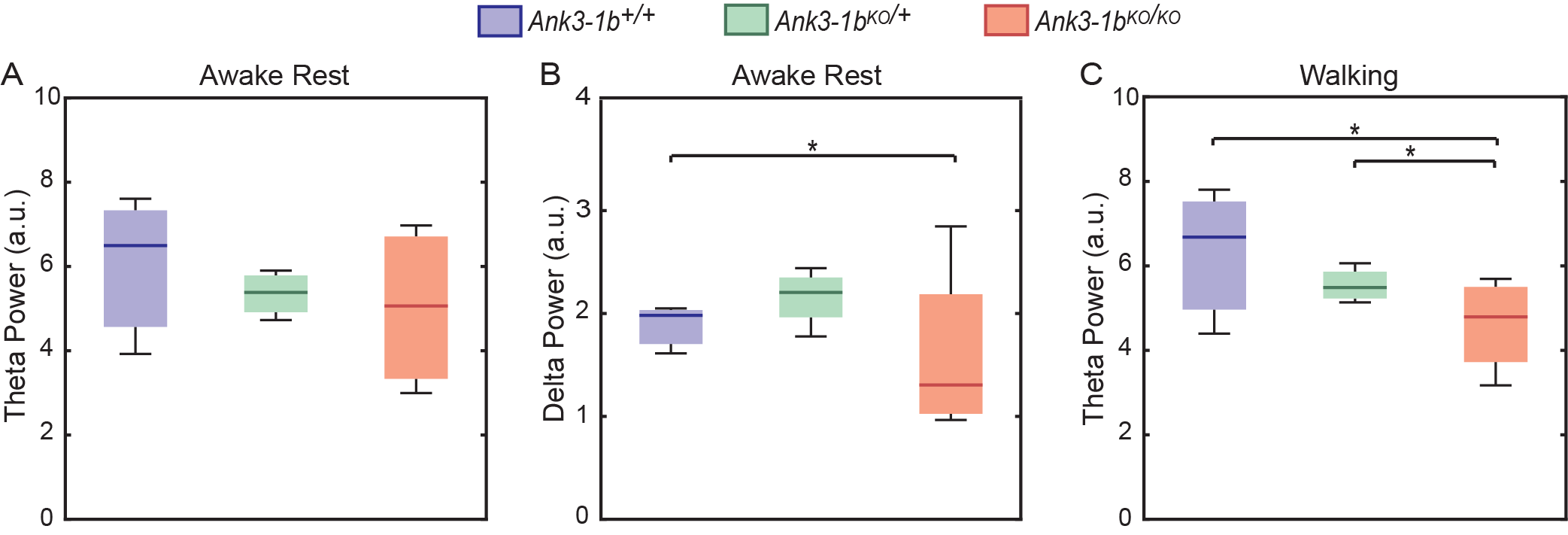


**Supplementary Figure 12**

**— *Ank3-1b^KO/KO^* mice have reduced delta and theta power during waking states. A:** Boxplots of estimated theta power (6-10 Hz) during awake rest showing no significant differences between genotypes (no significant main effect of genotype: F(2,53) = 1.7, p = 0.2). **B:** Boxplots of estimated delta power (2-5 Hz) during awake rest showing that *Ank3-1b^KO/KO^* mice have reduced delta power compared to WT(significant main effect of genotype: F(2,53) = 67.8, p < 0.001; post-hoc pairwise comparison: *Ank3-1b^KO/KO^* vs wildtype: t(53) = 3.2, *p < 0.005). **C:** Boxplots of estimated theta power (6-10 Hz) during walking showing that *Ank3-1b^KO/KO^* mice have significantly reduced theta power compared to WT and *Ank3-1b^KO/+^* mice (significant main effect of genotype: F(2,56) = 56.8, p < 0.001; post-hoc pairwise comparisons: wildtype vs *Ank3-1b^KO/+^*: t(56) = 9.4, *p < 0.001; *Ank3-1b^KO/KO^* vs wildtype: t(56) = 9.2, *p < 0.001).


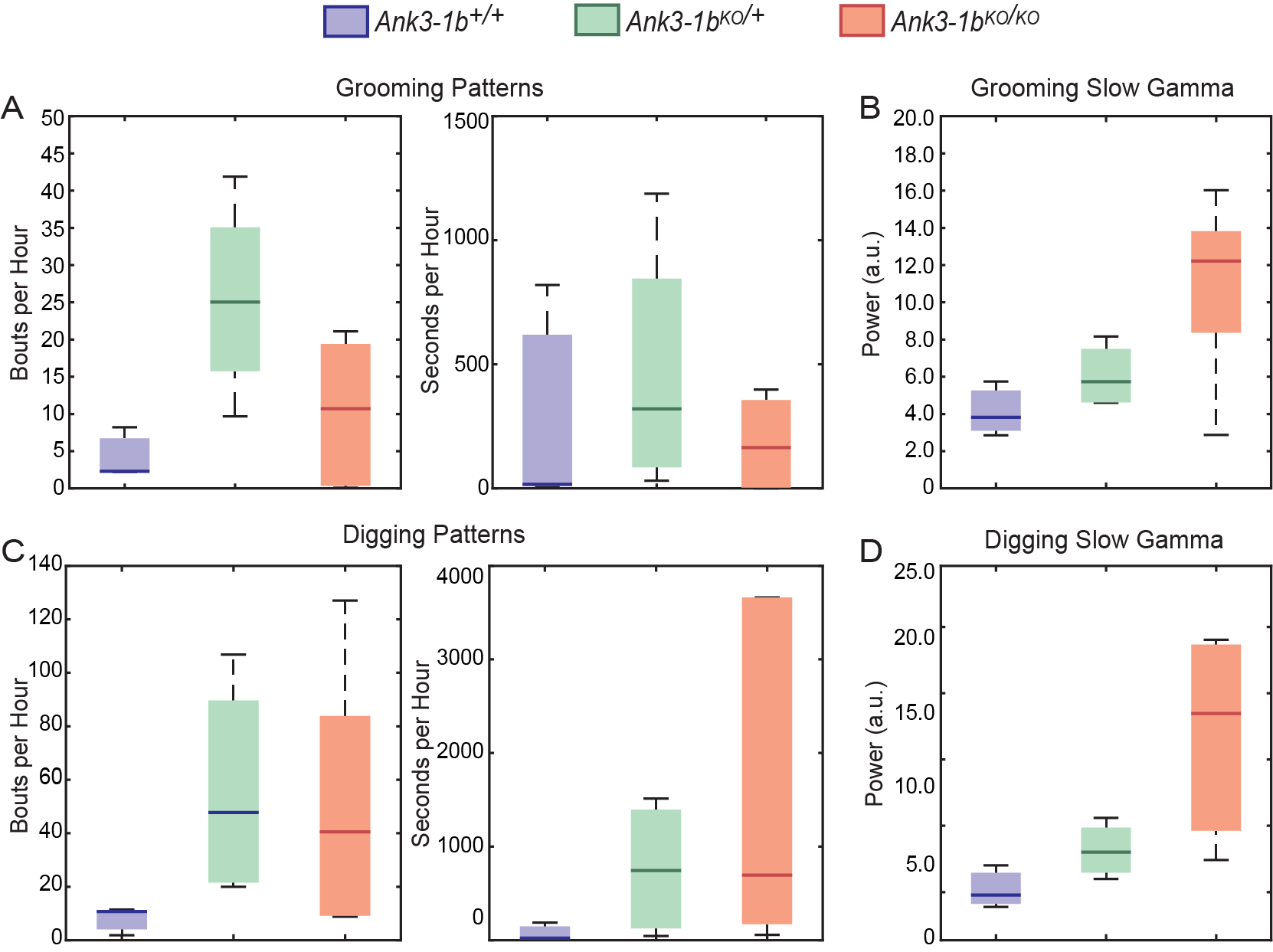


**Supplementary Figure 13—Distribution of individual mouse repetitive behaviors data.** **A:** Boxplots showing the distribution of average frequency of grooming bouts (left) and average time spent grooming (right) per (WT: n = 3 mice; *Ank3-1b^KO/+^*: n = 4 mice; *Ank3-1b^KO/KO^*: n = 5 mice). **B:** Boxplots showing the distribution of average estimated slow gamma power (25-45 Hz) for mice per genotype during grooming (WT: n = 3 mice; *Ank3-1b^KO/+^*: n = 4 mice; *Ank3-1b^KO/KO^*: n = 5 mice). **C:** Boxplots showing the distribution of average frequency of digging bouts (left) and average time spent digging (right) per hour (WT: n = 3 mice; *Ank3-1b^KO/+^*: n = 4 mice; *Ank3-1b^KO/KO^*: n = 5 mice). **D:** Boxplots of average estimated slow gamma power (25-45 Hz) for mice per genotype during digging (WT: n = 3 mouse; *Ank3-1b^KO/+^*: n = 4 mice; *Ank3-1b^KO/KO^*: n = 5 mice).


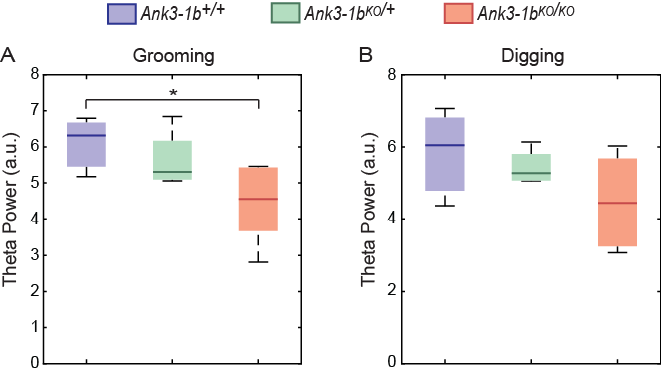


**Supplementary Figure 14— *Ank3-1b^KO/KO^* mice have reduced theta power during grooming. A:** Boxplots of estimated theta power (6-10 Hz) during grooming showing that *Ank3-1b^KO/KO^* mice have significantly reduced theta power compared to W10T mice (generalized linear mixed model, significant main effect of genotype: F(2,60) = 5.3, p = 0.008), but the only post-hoc pairwise comparison that was significantly different was the comparison of theta power in *Ank3-1b^KO/+^* mice and wildtype mice (t(60) = 3.2, *p = 0.006). **B:** Boxplots of estimated theta power (6-10 Hz) during digging showing that *Ank3-1b* mice have no difference in theta power compared to WT mice (generalized linear mixed model, significant main effect of genotype: F(2,68) = 1.3, p = 0.268).
